# The caste- and sex-specific DNA methylome of the termite *Zootermopsis nevadensis*

**DOI:** 10.1038/srep37110

**Published:** 2016-11-16

**Authors:** Karl M. Glastad, Kaustubh Gokhale, Jürgen Liebig, Michael A. D. Goodisman

**Affiliations:** 1 Department of Cell and Developmental Biology, University of Pennsylvania Perelman School of Medicine, Philadelphia, Pennsylvania, 19104, USA; 2 School of Biology, Georgia Institute of Technology, Atlanta, GA 30332, USA; 3 Department of Environmental Science Policy and Management, University of California, Berkley, 94720, USA; 4 School of Life Sciences, Arizona State University, Tempe, Arizona 85287, USA

## Abstract

Epigenetic inheritance plays an important role in mediating alternative phenotype in highly social species. In order to gain a greater understanding of epigenetic effects in societies, we investigated DNA methylation in the termite *Zootermopsis nevadensis*. Termites are the most ancient social insects, and developmentally distinct from highly-studied, hymenopteran social insects. We used replicated bisulfite-sequencing to investigate patterns of DNA methylation in both sexes and among castes of *Z. nevadensis.* We discovered that *Z. nevadensis* displayed some of the highest levels of DNA methylation found in insects. We also found strong differences in methylation between castes. Methylated genes tended to be uniformly and highly expressed demonstrating the antiquity of associations between intragenic methylation and gene expression. Differentially methylated genes were more likely to be alternatively spliced than not differentially methylated genes, and possessed considerable enrichment for development-associated functions. We further observed strong overrepresentation of multiple transcription factor binding sites and miRNA profiles associated with differential methylation, providing new insights into the possible function of DNA methylation. Overall, our results show that DNA methylation is widespread and associated with caste differences in termites. More generally, this study provides insights into the function of DNA methylation and the success of insect societies.

Phenotypic plasticity is a highly important mechanism whereby a single genotype can produce multiple phenotypes based upon environmental variation. Social insects represent excellent models for studying phenotypic plasticity. In insect societies, individuals can develop distinct social phenotypes (castes), usually through the integration of information from the environment^1^,^2^. Indeed, the production of castes is one of the major factors responsible for the ecological dominance of social insects^1^.

Phenotypic plasticity, such as that displayed in insect societies, requires epigenetic information. Epigenetic information is not coded in the standard complement of DNA bases but nevertheless results in heritable changes in gene expression^3^. One important and widespread form of epigenetic information is the methylation of DNA^4^,^5^ which has been identified as a functional epigenetic mechanism in diverse eukaryotes including plants, animals, and fungi^5^. Consequently, DNA methylation represents an extremely important form of epigenetic information across eukaryotic systems.

DNA methylation has been implicated as an important component of the determination of social insect caste^6^. Indeed, knockdown of the putative mediator of *de novo* DNA methylation (DNMT3) was shown to have a direct impact on the production of castes in the honey bee^7^. DNA methylation has further been associated with alternative splicing differences between honey bee castes^8^,^9^, linked to modulation of context-dependent gene expression^10^, and found to display differences between castes in hymenopteran social insects^11^,^12^.

However, the function of DNA methylation in social insects remains controversial^6^,^9^,^13^. Indeed, recent studies have questioned whether previously implemented statistical approaches robustly support meaningful differences in DNA methylation among social insect castes^14^. Thus the questions of whether DNA methylation is associated with the generation of social phenotypes, and how such associations may vary among species, remain unclear. Notably, a major gap in our understanding of DNA methylation arises because almost all prior work on epigenetic effects in social insects has been conducted in the social Hymenoptera (ants, social bees, and social wasps)^15^,^16^.

Termites (epifamily Termitoidae) represent an entirely novel origin of sociality in insects, and are distinct from the Hymenoptera in many ways^17^. Termites evolved from wood-dwelling roach-like ancestors^18^ and separated from the hymenopteran social insects over 350 MYA^19^. Termites also possess a hemimetabolous system of development, whereby juveniles become more and more adult-like at each of multiple molts. This differs greatly from the holometabolous hymenopteran insects where the final larval instar goes through metamorphosis during the pupal stage, resulting in drastic morphological changes ultimately leading to the adult form. Thus, the developmental program that underlies termite societies differs substantially from that found in social Hymenoptera. In lower termites, for example, workers are composed of multiple, developmentally progressive instars. Furthermore, final worker instars are poised to develop into either soldiers, winged reproductives, or neotenics, which are a specialized worker-derived reproductive form^17^,^20^,^21^.

Termite developmental plasticity is largely informed by hormonal (endogenous) and environmental (exogenous) cues^22^,^23^,^24^. Moreover, termite castes are arguably more protean than those in the social Hymenoptera. Termites further differ from hymenopteran social insects in that both sexes are often represented in all castes in the colony (e.g. ref. 25), allowing for an examination of caste differences whilst controlling for sex. Moreover, preliminary research indicates that termites possess a functional suite of DNA methyltransferase enzymes, as well as putatively high levels of DNA methylation in their genome^21^,^26^, suggesting the possibility that DNA methylation may have important functions in termites. Thus, termites are an enticing system for studying the molecular mechanisms underlying caste differentiation and development, and provide an under-studied alternative to the hymenopteran social insects.

In order to gain a greater understanding of the function, evolution, and importance of epigenetic information in social insects, we investigated the distribution and levels of DNA methylation and gene expression in both sexes of the final larval instars (workers) and winged reproductives (alates) of the termite *Zootermopsis nevadensis*. This species is common in the cooler coastal and higher elevations of the western USA^27^. It belongs to the lower termites that live inside of wood and that do not forage outside. This type of social organization is associated with little or no brood care by workers^28^. However, workers of *Z. nevadensis* frequently engage in anal feeding as part of their social interactions. Workers eventually develop into either alates, neotenic reproductives or the altruistic soldiers^21^. Alates leave the colony, mate and found a new colony as a queen and king pair. When the queen or the king dies, they are replaced by neotenics that mate with their brothers or sisters. Older colonies of *Z. nevadensis* therefore show different levels of inbreeding depending on the number of replacement reproductives. *Z. nevadensis* thus represents an interesting termite system in which to study DNA methylation in the context of sociality.

Overall, we found that DNA methylation was considerably higher and targeted to more genes in termites than in any other social insects studied to date. Moreover, patterns and levels of DNA methylation were more similar to non-insect invertebrates than to other previously-analyzed holometabolous insects. We also found that termite morphs displayed many differences in DNA methylation, the great majority of which occurred between castes. Differential methylation was also related to alternative splicing, and showed enrichment for multiple regulatory sites. Thus, our results provide insight into the evolution and regulatory function of DNA methylation in insects and expand our understanding of DNA methylation’s link to phenotypic plasticity in insect societies.

## Results

### Termite genes are highly methylated

We sequenced the methylomes and transcriptomes of male workers, female workers, male alates, and female alates (AM, AF, WM, WF) from a single *Z. nevadensis* termite colony. Methylome sequencing was replicated twice, and transcriptome sequencing was replicated three times, for each sample type (8 total methylomes, 12 total transcriptomes). This provided four replicates for comparisons of DNA methylation between caste and four replicates for comparisons of DNA methylation between sexes. Methylomes and transcriptomes were derived from whole body minus guts. Methylome data were obtained from pooled individuals, whereas transcriptome data were obtained from individual termites.

**Figure 1:**
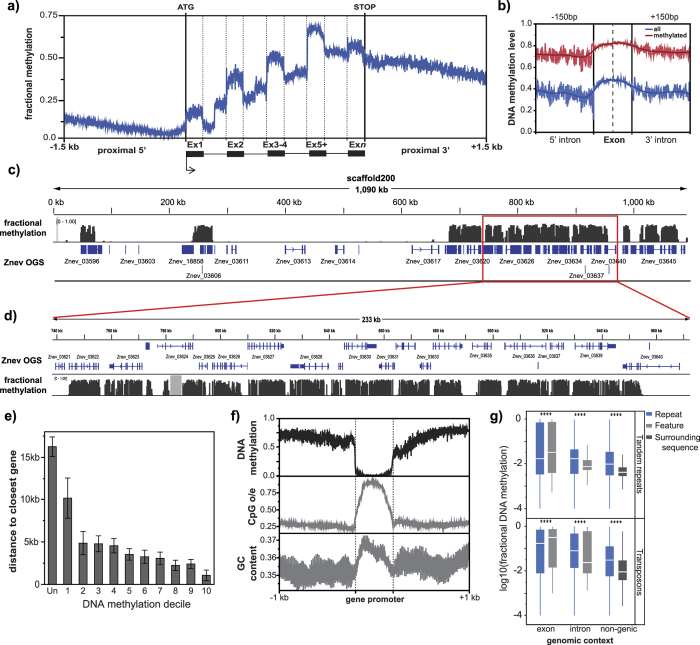
Genome wide DNA methylation patterns in *Z. nevadensis*. (**a**) Methylation profile across multi-exon genes in *Z. nevadensis*. (**b**) Spatial profile of DNA methylation at internal exon-intron junctions across all exons (blue) and methylated exons only (red). (**c**) Genome browser snapshot of scaffold 200 and (**d**) 233 kb subset of scaffold 200, illustrating highly methylated gene-dense regions commonly seen in *Z. nevadensis*. Gray box indicates missing data. (**e**) Genomic distance to the nearest adjacent gene for 10 deciles of ascending DNA methylation level (1–10) as well as for unmethylated (Un) genes (error bars represent 95% CIs). (**f**) Spatial plot of mean fractional DNA methylation, CpG o/e and GC content within and around unmethylated promoters of methylated genes. (**g**) Mean DNA methylation level of repeats possessing three or more CpGs organized by repeats intersecting exons, introns, and non-genic repeats, and mean methylation level of the intersecting feature or adjacent surrounding sequence. ****Significant (P < 0.0001) difference between repeat and surrounding feature or sequence.

We found exceptionally high levels of DNA methylation in termites (Figs 1 and S1). Over 12% of genomic CpGs and 58% of exonic CpGs were methylated when considering data pooled across all samples (Table S4). The average methylation fraction (percentage of methylation) across all exonic CpGs was 44.1%. In addition, over 70% of genes featured significant methylation targeted to one or more exons (77.6% of genes as exons + introns). Levels of DNA methylation showed good agreement with previously-published measures of CpG depletion (r = −0.635, p < 0.0001; ref. 21). Among genes methylated in our pooled data, the majority (88.5%) were methylated in all samples, with only 5.1% methylated in less than half (4/8) of our samples. We also found that termites displayed considerable methylation throughout genes, with DNA methylation increasing from 5′ → 3′ within the gene body (Figs 1a,c and S2). We further observed that regions downstream of methylated genes exhibited considerable methylation (Figs 1a,c,d and S2,S3). This contrasts with the distribution of DNA methylation in hymenopteran insects, where DNA methylation is preferentially targeted near gene starts^16^.

DNA methylation was targeted to both exons and introns in *Z. nevadensis*, with considerable methylation in introns (Figs 1a,b and S1). Indeed, although a higher proportion of CpGs were methylated in exons, over twice as many mCGs existed within introns because introns are considerably larger than exons (Table S4). However, the difference between exon and intron methylation was low when limited to exons for which data on both up- and down-stream introns were available (Fig. 1b). Overall, this result is in stark contrast to that seen in holometabolous insects, where exons show much higher levels of methylation than introns^11^,^15^,^16^.

We found that methylated and unmethylated gene sets were enriched for particular functions (Table S7). Specifically, methylated genes were most highly enriched for functional terms related to fundamental cellular processes (e.g. ATP binding, DNA repair, histone modification), while unmethylated genes were associated with terms associated with more developmentally- or temporally-regulated genes linked to organismal development (e.g. odorant binding, development of primary sexual characteristics, *Wnt* signaling pathway). We found that the most highly methylated genes (top 3 DNA methylation deciles; 3,072 genes) showed functional enrichment of terms associated with more fundamental regulatory processes such as chromatin modification, protein binding, helicase activity, metabolic processes, and regulation of gene expression, while the most lowly methylated genes (bottom 3 DNA methylation deciles) were functionally enriched for more dynamic terms such as “signaling receptor activity”, transmembrane transport of various molecules, cell periphery, and circadian behavior (Table S8). These general associations are similar to those seen in other insects. Thus, despite the stark differences in levels and intragenic patterning of DNA methylation in termites and holometabolous insects, the types of genes targeted by DNA methylation are conserved across insects.

### Regions of vertebrate-like methylation within the *Z. nevadensis* genome

The majority of methylated CpGs in *Z. nevadensis* exhibited high levels of DNA methylation, with 87.5% of methylated CpGs possessing >50% methylation fraction (Fig. S1). However, a minority of CpGs displayed low levels of methylation or were essentially unmethylated (Fig. S1). Similarly, genes tended to fall into two distinct classes, where some genes were essentially unmethylated and others were highly methylated (Figs 1 and S1).

We found that methylated genes tended to be clustered together within the *Z. nevadensis* genome, and were much more closely-spaced than unmethylated genes (Figs 1d and S3). Indeed, clusters of methylated genes resulted in large regions of highly-methylated CpGs, wherein regions up to hundreds of kilobases in size possessed high levels of DNA methylation at the majority of CpG sites (Figs 1c,d and S3). Within such methylation “blocks” in termites, the great majority of unmethylated CpGs corresponded to gene promoters (Fig. 1d,f). Furthermore, due to the increased evolutionary mutability of methylated cytosines, within such regions, CpG densities were depleted everywhere but within gene promoters (Fig. 1f). This results in such regions showing intriguing similarities to patterns of methylation in vertebrates, where CpGs are methylated everywhere but within gene promoters.

We found that several classes of DNA repeats were methylated, as is the case in vertebrate systems. However, the interpretation of this finding in termites was complicated by the fact that many repeats fell within introns, which were often highly-methylated in *Z. nevadensis*. We thus examined genic repeats (those intersecting exons or introns) and non-genic repeats separately. We found that, a low proportion of non-genic repeats showed at least some DNA methylation, which was higher within non-genic-repeat elements than in surrounding regions (Figs 1g and S4). Interestingly, genic repeats intersecting exons appeared to have lower levels of methylation than the exon they fell within (Wilcoxon rank-sum pvalue: <0.0001; Fig. 1g). However, the majority of gene-intersecting repeats fell within introns, where repeats were more highly methylated than the containing intron (Figs 1g and S4). Despite this, we found that for both methylated exons and introns, those containing repeats were, in general, less methylated than those without (Fig. S4). Thus, some repeats did appear to be methylated in *Z. nevadensis*, although the level and prevalence of repeat methylation is lower than seen in vertebrates.

### Conservation of methylation in orthologous genes of social insects

Unlike many insects examined to date, the *majority* of *Z. nevadensis* genes are methylated (74% methylated in at least one sample, 66% methylated in all 8 samples), and at higher levels (but see ref. 29). Moreover, 12% of genomic CpGs and 58% of exonic CpGs were methylated in *Z. nevadensis* (at least two samples; Table S4 and Fig. S1). In contrast, only 1–2% of genomic CpGs are methylated in other social insects^15^. We thus contrasted DNA methylation in *Z. nevadensis*, where ~75% of genes are methylated, with DNA methylation in hymenopteran social insects, where ~35% of genes are methylated (*A. mellifera*: 38.2% 4,946/12,961, *C. floridanus*: 35.7% 5,538/15,510).

**Figure 2:**
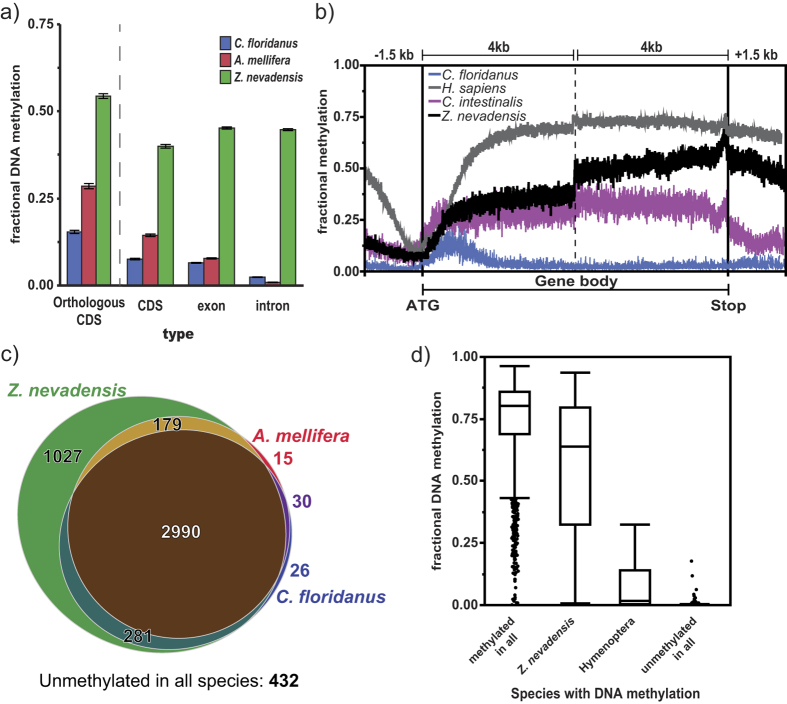
DNA methylation in *Z. nevadensis* exists at higher levels and is targeted to more genes than in hymenopteran social insects. (**a**) Fractional methylation for coding sequences among conserved 1-to-1 orthologs between *C. floridanus, A.mellifera*, and *Z. nevadensis* (Orthologous CDS), all coding sequences (CDS), exons, and introns (error bars: 95% confidence inverval of mean). (**b**) DNA methylation levels within the first and last 4 kb of gene bodies (exons + introns) for *Z. nevadensis* (black), for a representative hymenopteran (blue), non-insect invertebrate (*Ciona intestinalis*; purple), and mammal (*Homo sapiens;* grey). (**c**) Venn diagram of methylation status of 4,931 conserved 1-to-1 orthologs showing large number of genes methylated only in *Z. nevadensis*. (**d**) Mean fractional DNA methylation levels for genes classified based upon methylation status of the same orthologs and species as in ‘c’.

Overall, we found that almost all genes that were methylated in the *C. floridanus* and *A. mellifera* were also methylated in *Z. nevadensis* (only 71 of 5,019 shared orthologs were methylated in ants and bees but not in *Z. nevadensis*, Figs 2c and S6). In contrast, there were many genes methylated in *Z. nevadensis* that were unmethylated in *C. floridanus* and *A. mellifera* (1027/5,019 shared orthologs; Fig. 2c). Genes methylated in *Z. nevadensis*, but not *C. floridanus* or *A. mellifera*, were highly enriched for terms relating to tissue- or temporal-specific gene expression (Table S9). This result is consistent with the fact that highly-methylated genes in the Hymenoptera are primarily ubiquitously-expressed housekeeping genes. Thus, for example, genes methylated only in *Z. nevadensis* showed greater than 10-fold enrichment for many terms, including rhythmic process, sensory perception of chemical stimulus, and growth factor activity (13.6, 14.8 and 22.16 fold enrichment, respectively). Moreover, despite the lineage specific methylation of these genes, their mean methylation level was considerable (median methylation fraction: 0.64, Fig. 2d) and reached methylation levels similar to other methylated genes in the *Z. nevadensis* genome.

### DNA methylation is associated with gene expression

**Figure 3:**
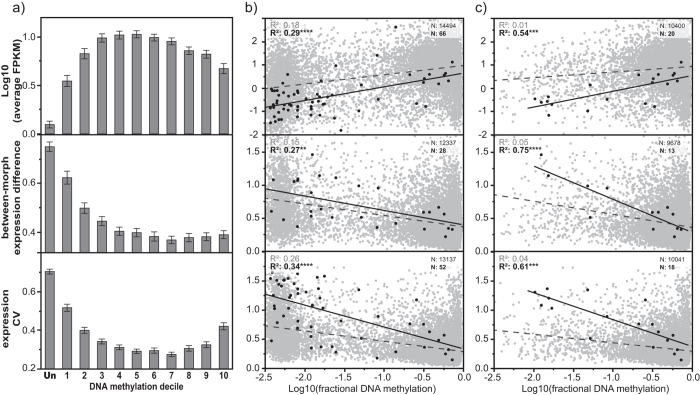
The relationship between DNA methylation and gene expression in *Z. nevadensis*. (**a**) Gene expression level, between-morph absolute expression difference, and within-morph replicate coefficient of variation (CV) for deciles of increasing DNA methylation (1–10) and unmethylated genes (Un; error bars: 95% confidence interval of mean). (**b**) Regression of expression metrics against a continuous measure of DNA methylation among all genes, divided into genes having undergone recent duplication (black, solid line), and those that have not (grey, dashed line). (**c**) The same as in (**b**) but for methylated genes only. ****p < 0.0001, ***p < 0.001, **p < 0.01, *p < 0.05.

We found that levels of DNA methylation were positively associated with gene expression level (rho: 0.348, R^2^: 0.179, p: <0.0001). However, genes of intermediate methylation level were the most highly, and ubiquitously expressed (Fig. 3a). Level of DNA methylation was also negatively correlated with expression variance between samples (rho: −0.356, R^2^: 0.155, p: <0.0001) and within-morph replicate variation (CV; rho: −0.449, R^2^: 0.279, p: <0.0001; Fig. 3b). Thus genes showing variation in expression across samples tended to show low levels of methylation.

In order to isolate the strongest predictors of DNA methylation in termites, we performed regression analysis between DNA methylation level and gene expression variables (i.e., gene expression level, coefficient of variation, specificity), as well as intergenic distance, average exon count and general measures of gene conservation. We also leveraged the stranded nature of our RNA-seq protocol to produce a measure of anti-sense expression, which was incorporated into our combined model.

We found the strongest predictors of levels of DNA methylation in the combined model framework were expression CV and between-sample expression difference, which were both strongly negatively associated with genic DNA methylation level (Fig. S5). Interestingly, we also found that the level of antisense gene expression was more strongly associated with DNA methylation level than gene expression level from the sense strand (Fig. S5). This suggests that DNA methylation’s association with gene expression level and variation may be driven, at least in part, by an interaction between DNA methylation and intragenic antisense transcription^10^,^30^.

We next examined DNA methylation’s relationship with gene expression among recently-expanded gene families^21^. We found gene expression level and expression breadth were much more strongly associated with DNA methylation level among recently-duplicated genes with at least one methylated copy than among all genes (Fig. 3b). Furthermore, when considering only methylated genes, the relationship between DNA methylation level and gene expression variables was even stronger among methylated genes that were recently duplicated, despite the strong reduction in association when considering all (non-duplicated) methylated genes (Fig. 3c).

### Castes show strong differences in DNA methylation

Overall, we found that a very high proportion of genes possessed regions differentially methylated between phenotypes (castes or sexes). In total, 2,749 genes (of 10,974 tested genes) possessed significant differentially methylated regions (DMRs), defined as 200 bp windows showing significant differential methylation, between phenotypes. Interestingly, relatively few genes were differentially methylated between sexes (368 genes). Instead, the vast majority of differentially methylated genes (DMR-containing genes: DMGs) arose between the reproductive and worker castes (2,720 genes; Table 1, Figs S5 and S7). Even more surprisingly, we found that the great majority of these caste-DMGs exhibited higher methylation in alates than workers (Table 1). Thus, the great majority of significant differences in DNA methylation between phenotypes consisted of genes exhibiting higher methylation in the alate caste.

Recently, the importance of DNA methylation in the determination of caste has been called into question, with many early genome-wide studies of DNA methylation’s relation to caste differentiation being criticized for their lack of replication^14^. In order to address this issue in our data, we compared the observed number of DMRs between castes and sexes to the number of DMRs observed when replicates were randomly shuffled. Our results from this analysis show that the number of observed differences in DNA methylation between castes was far higher than expected by chance (Fig. S8). On the other hand, the number of sex differences observed were only marginally higher than that expected by chance. Based upon these tests we find strong evidence for caste differential DNA methylation, further supporting its association with termite caste in our data.

DMGs were enriched for multiple functional categories related to development and environmental responsiveness (Table S11). For example, multiple development-associated terms showed greater than two-fold enrichment among DMGs (e.g. embryonic pattern specification, motor activity, regulation of Rho signal transduction; Table S11). Moreover, several terms showed greater than five-fold enrichment among DMRs (double-stranded RNA binding, regulation of cell projection organization, and GTPase binding; Table S11).

We observed that DMGs showed a signal of increased expression in the morph of hypermethylation (Fig. S9). However, this association was weak and not significant in every instance. Furthermore, DMGs were significantly less variably expressed between replicates of the same morph, and showed less *absolute* expression difference between morphs when compared to genes that were not differentially methylated (Fig. S10). We further observed that DMGs were more likely to be conserved across insects than methylated genes. DMGs were also less likely to be duplicated when compared to methylated genes that did not contain DMRs (Fig. S10).

**Figure 4:**
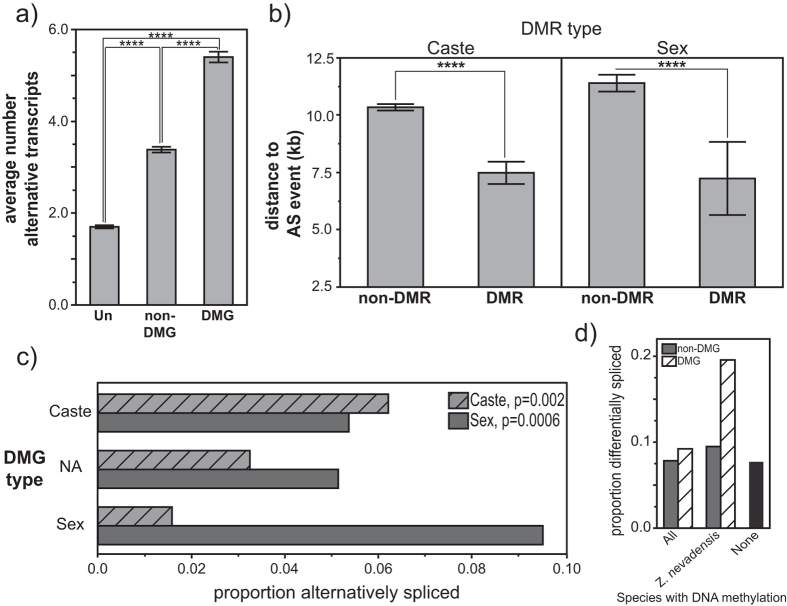
Differentially methylated genes show higher levels of alternative splicing. (**a**) Number of alternative isoforms observed among unmethylated genes (Un), non-differentially methylated genes (non-DMG), and differentially methylated genes (DMG). (**b**) Distance to the nearest differentially-spliced exon for non-differentially methylated regions (non-DMR) and differentially methylated regions (DMR) for both caste- and sex-DMGs. (**c**) Proportion of genes showing alternative splicing between castes and sexes for genes differentially methylated between castes, sexes, or neither (NA) caste nor sex (p-values from fishers exact test). (**d**) Proportion of DMGs and non-DMGs featuring a differential splicing event among genes methylated in *C. floridanus, A. mellifera*, and *Z. nevadensis* (All), methylated only in *Z. nevadensis*, and unmethylated in all three species (as in Fig. 2c). Error bars: 95% confidence interval of mean.

Notably, DMGs were more likely to feature at least one alternatively spliced variant than unmethylated genes or methylated genes without DMRs (Fig. 4a). Interestingly, the proportion of genes significantly differentially spliced between caste or sex was significantly higher for genes containing caste- or sex-specific DMRs, respectively (Fig. 4c). That is, differential methylation between phenotypes was associated with alternative splicing differences between the same phenotypes. We further found that DMRs were located significantly closer to differentially-spliced exons than nonDMRs within differentially spliced DMGs (Fig. 4b). Finally, we found that genes methylated only in *Z. nevadensis* and not in ants or bees showed higher overall levels of alternative splicing, when compared to genes methylated in all species. Indeed, DMGs methylated only in *Z. nevadensis* showed almost two-fold more alternative splicing than non-DMGs (Fig. 4d). Therefore, overall, there was a significant and notable relationship between differential methylation of genes and alternative splicing in *Z. nevadensis*.

### Enrichment of regulatory motifs surrounding differentially methylated cytosines (DMCs)

We sought to evaluate if regions of the *Z. nevadensis* genome that were differentially methylated between phenotypes showed significant over-representation of transcription factor binding sites (TFBSs). We first performed statistical tests examining the relative enrichment of existing *D. melanogaster* TF motif profiles (flyreg^31^ and idmmpmm^32^ profiles) within sequences centered on confidently differentially methylated cytosines (DMCs), relative to nearby sequences not showing significant differential methylation. Importantly, our control sequences were centered on methylated cytosines that did not differ between phenotypes (non-DMCs), in order to control for composition biases associated with CpG-centered sequences and CpG depletion commonly found in methylated regions.

We found that DMCs exhibited significant enrichment for multiple TFBS motifs including those related to several key developmental TFs with existing orthologs in *Z. nevadensis*. While the majority of these TFBSs were enriched only among caste-specific DMCs, several TFBSs were enriched surrounding sex-specific DMCs. Two of these putative binding sites, for TFs *zeste* and *nubbin*, showed enrichment only for sex-specific DMCs, as well as relative to when using caste-specific DMCs as control regions (Table 2, S13).

We also performed *de novo* motif identification (MEME) to identify any enriched motifs in the vicinity of DMCs, independent of any *a priori* motif sets. We identified several such motifs in the *Z. nevadensis* genome. The majority of identified motifs were long (>15 bp) and exhibited considerable similarity to known *D. melanogaster* miRNAs (Table S14). We thus utilized putative mature miRNA sequences from *Z. nevadensis*^21^ to examine the enrichment of miRNAs surrounding DMCs.

We found that multiple miRNA-like sequence motifs were likely to be found within DMCs relative to control sequences (Table 3 and S15). We found that, in the great majority of cases, only one of the two mature miRNAs produced from the putative associated miRNA hairpin showed significant overrepresentation among DMCs. For example, all 267 hits for the miRNA zne-mir-6012 were to the 5-prime mature miRNA, whereas there were no hits to the 3-prime miRNA (Table S15). miRNAs have classically been most strongly implicated in post-transcriptional silencing, through binding to 3′UTRs of mRNAs. Thus we examined the genic distribution of our miRNA-hitting DMCs, expecting many to fall within the putative 3′UTR of the target genes. Interestingly, we found that the majority of DMCs that featured at least one significant mature miRNA motif fell within exons or introns (Table S16; ~86%); only ~8.5% fell within 2 kb downstream of a given gene model. Thus, the majority of DMC-surrounding sequences that feature significant similarity to miRNAs fall within gene bodies and not 3′UTRs.

## Discussion

Our results suggest DNA methylation is targeted to many more *Z. nevadensis* genes than in the hymenopteran social insects. Moreover, the distribution of DNA methylation across *Z. nevadensis* gene bodies is more similar to that seen in the invertebrate *C. intestinalis* than to the holometabolous insects^33^. The phylogenetic distribution of well-characterized DNA methylomes is highly biased to holometabolous insects^15^, and preliminary evidence in several hemimetabolous insects suggests that DNA methylation may exist at higher levels in hemimetabolous insects in general^16^,^21^,^26^,^29^,^34^. Thus, the higher levels of methylation seen in termites may reflect an ancestral loss of DNA methylation in the Holometabola, and not an expansion of methylation in *Z. nevadensis*. Nevertheless, the strong functional enrichment of genes methylated only in *Z. nevadensis* suggests that genes methylated solely in *Z. nevadensis* show greater developmentally or temporally-regulated expression than genes methylated in both Hymenoptera and termites.

We also observed that methylated genes were often clustered together within the *Z. nevadensis* genome. These highly methylated regions were punctuated by unmethylated CpGs, which often corresponded to gene promoters, resulting in the emergence of CpG-enriched promoters, surrounding by CpG-depleted, highly-methylated regions (Fig. 1). The lack of methylation of such promoters bears striking resemblance to the CpG islands seen in vertebrates^35^, raising the intriguing possibility that DNA methylation in termites provides a glimpse at an ancestral state in the evolution of vertebrate CpG islands.

DNA methylation was strongly and negatively associated with expression noise (between-replicate expression variance). Interestingly, previous studies, even in mammals, have observed a negative relationship between intragenic DNA methylation and gene expression noise^16^,^36^,^37^, suggesting a conserved function of DNA methylation may be to suppress transcriptional noise.

Strikingly, we found the relationship between DNA methylation and gene expression level or breadth to be much stronger among gene families having undergone recent duplication. This suggests that DNA methylation may play a novel role in regulating the expression of recently-duplicated genes^38^. These observations are in agreement with at least one study in honey bees where DNA methylation was observed to regulate the neofunctionalization of a duplicate gene, supporting a role for DNA methylation in contributing to functional diversification of duplicate genes^39^. Alternatively, in our data, DNA methylation may simply correlate better with gene expression within rapidly sub-functionalizing gene duplicates due to the duplicate’s rapid loss of function.

We compared replicated DNA methylation between males and females and between reproductive and worker termite castes. We found substantial differences in methylation between castes, and to a lesser degree sexes, demonstrating that developmental phenotypes of termites do display methylation differences. We assayed DNA methylation at the whole organism level. Therefore, the methylation differences we uncovered may reflect allometric variation between castes or sexes arising from whole body analyses. Thus further analyses of tissue-specific methylation would be of interest and help in ascertaining the exact tissues causing the differences between termite phenotypes. In addition, analyses of time matched samples would help isolate the effects of age on DNA methylation, which may also be a contributing factor to the methylation differences we observed. We also note that our analyses were centered on a single *Z. nevadensis* colony. Thus further analyses of DNA methylation from more colonies would help determine if methylation differences are affected by genetic and environmental variation arising from colony differences.

Our finding of differential methylation between social insect phenotypes stands in contrast to recent studies that did not detect methylation differences between castes^14^. However, analyses of false discovery in our data strongly support the existence of methylation differences between termite castes (Fig. S8). It is possible that the behavioral plasticity arising from individuals oscillating between reproductive and non-reproductive states^14^ may not be associated with DNA methylation differences. In contrast, the more permanent changes in phenotype observed here may be associated with methylation differences. That is, irreversible developmental canalization, as studied in this investigation, may be associated with methylation differences whereas reversible behavioral switching may not. More generally, the effects of DNA methylation on caste differentiation may differ among species. Regardless, it is clear that more careful analyses is required to determine if methylation differences actually do exist between social insect castes and if the observed levels of caste-biased methylation reflect differences between termites and hymenopteran social insects or are representative of adaptations in juveniles versus adults.

We found that the majority of differences in DNA methylation existed between castes, with far fewer existing between sexes. Both caste- and sex-specific differentially methylated genes (DMGs) were less variably expressed, but showed considerably higher levels of alternative splicing than non-DMGs. Importantly, we also found that both caste- and sex-DMGs exhibited higher proportions of *differential* splicing between castes and sexes, respectively. Notably, differences in DNA methylation in bees and ants have been linked to differences in alternative splicing, and it is hypothesized that this relationship may underlie DNA methylation’s impact on caste determination in social insects^8^,^9^,^11^. Thus, our results suggest that the connection between alternative splicing and differential methylation may be a broadly-conserved mechanism linking alternative phenotype to differential methylation in insects. The connection between alternative splicing and differential methylation may be stronger in termites than other insects, however, as we found that differential methylation was more strongly associated with alternative splicing in genes methylated in *Z. nevadensis* only but not Hymenoptera.

Gene body DNA methylation differences have been experimentally implicated in the regulation of differential splicing in mammals^40^,^41^. In most cases, DNA methylation differences impact splicing through an alteration of a transcription factor’s (TF) binding accomplished either directly through methylation’s impact on TF binding^40^,^42^,^43^ or by altering local chromatin resulting in changes to DNA accessibility^41^,^44^. Indeed, these same mechanisms are thought to underlie the observed strong negative association between mammalian promoter methylation and expression level of the associated locus^5^, as methylated promoters are associated with a less accessible chromatin state and an inability to initiate transcription^45^,^46^. Furthermore, at least one study has implicated DNA methylation in modulating context-dependent expression of target genes, possibly through an interaction with antisense transcription^10^.

Interestingly, we found that multiple transcription factor binding site (TFBS) motifs were associated with differential methylation in *Z. nevadensis*, suggesting that methylation is linked to alterations in TF binding in termite genes. We further found that many of these binding sites exhibited enrichment in regions differentially methylated only between caste or sex. Notably, many of these enriched TFBSs were associated with developmental processes. Therefore, in termites, differential methylation is targeted to many regions potentially bound by important regulators of development. Thus DNA methylation may represent an important evolutionarily-conserved molecular mechanism that impacts developmentally-important TF binding.

Surprisingly, we also found multiple mature miRNA or miRNA-like sequences associated with differentially-methylated cytosines of the *Z. nevadensis* genome. Although the canonical role of miRNAs is the regulation of translation, emerging evidence suggests that miRNAs can also impact the epigenome as well as the process of transcription^47^,^48^,^49^,^50^. In addition, several components of the RNAi pathway have been shown to associate with euchromatin in *Drosophila* in a smRNA-guided manner^51^. Thus, our results suggest DNA methylation may also play a role in altering the binding of regulatory RNAs in termites. This is bolstered by the observation that genes in differentially methylated regions that contain miRNA motifs were considerably enriched for multiple developmentally-associated functions (Table S17). This further suggests that DNA methylation’s interaction with miRNA binding is functionally important to insect development. Typically, miRNA genes produce two complementary mature miRNA templates; however, for the majority of miRNAs, only one of these two templates is utilized by the components of the RNAi pathway^52^. Thus, the fact that most of these focal miRNAs were only enriched for one of each pair of mature miRNAs supports the functional role of this association. Overall, the enrichment of specific regulatory motifs surrounding regions of differential methylation may reflect a major functional role for differential methylation in the phenotype-specific alteration of regulatory binding. This may, in fact, be one of the major ways in which DNA methylation impacts gene expression^43^,^46^,^53^.

Many of the TF and miRNA motifs significantly associated with variation in termite methylation have developmental functions in *D. melanogaster.* For example, two of the top TFs (Eip74EF and forkhead), as well as one miRNA (miR-125), in this category are directly associated with the regulation of ecdysone in *D. melanogater*^54^. Ecdysone is an important molting regulatory hormone in insects and implicated in the regulation of caste in both hymenopteran social insects and termites^21^,^55^. It is possible that this enrichment of regulatory sites within DMRs is a novel (or expanded) association, and is related to the higher level of DNA methylation in termites. Indeed, a great many methylated or differentially-methylated CpGs are found within introns and other non-coding gene-proximal sequences in *Z. nevadensis* (Table S6), which are generally unmethylated in other insects with DNA methylation. Although the observed relationship between DNA methylation and the putative motifs is suggestive of an important role for DNA methylation in gene regulation, it will be important to validate these results experimentally in order to determine if DNA methylation does, in fact, affect the action of TFs or miRNAs, particularly given the high level of divergence between termites and flies.

In conclusion, our study revealed that (i) levels of DNA methylation were much higher in termites than in the majority of other insects, (ii) DNA methylation was associated with gene expression, (iii) many significant differences in methylation existed between castes but few between sexes, (iv) caste and sex specific differentially-methylated genes showed higher levels of alternative splicing differences, and (v) regions surrounding differentially methylated cytosines were enriched for multiple regulatory motifs. Thus our study greatly increases the current knowledge of DNA methylation’s relationship to alternative phenotype across insects, and further provides a possible mechanism whereby DNA methylation may effect differential gene regulation in insects. Finally, our results highlight the general utility of termites as a developmental and evolutionary contrast to hymenopteran social insects, and illustrate termites as an excellent model for future molecular studies of epigenetic underwriting of insect caste and development.

## Methods

### Biological samples

A colony of *Zootermopsis nevadensis nuttingi* was collected in its entirety within a wood log from Pebble Beach near Monterey, California, United States in October 2013. The colony was kept in the original log in a large bin in the laboratory. The log was sprayed with water once per week and kept at 22˚ Celsius with a 12 h light/dark cycle. Species identity was confirmed by analyzing the cuticular hydrocarbon profiles from workers using gas chromatography-mass spectrometry^56^,^57^.

Individual male and female alates and workers (final instars) were collected for nucleic acid extraction five months following the original collection date. Individuals for RNA extraction were dissected in RNAlater, while individuals for DNA extraction were dissected in distilled water. The guts of all samples were carefully dissected out under a microscope and not included in nucleic acid analyses. Wings were removed from alates as well. Sex was determined based on external abdominal morphology.

### Nucleic acid isolation and sequencing

Total RNA was extracted from single individual termites in 1 ml of Trizol^®^ Reagent (Life Technologies) according to manufacturer’s instructions. The extracted RNA was suspended in 40 μL Nuclease Free Water (Ambion) and was treated with TURBO DNA-*free*™ kit (Ambion) to remove the genomic DNA contamination. RNA concentration was measured by Qubit ^®^2.0 fluorometer (Life Technologies, Carlsbad) and RNA integrity was checked by Agilent RNA 600 nano kit (Bioanalyzer, Agilent Technologies).

One termite was used per sequencing library for each RNA-seq sample and five termites were used per sequencing library (except for male alates, where only four individuals were used) for each BS-seq sample. Genomic DNA was isolated using MasterPure™ Complete DNA and RNA Purification Kit (Epicentre). The extracted DNA was suspended in 35 μL TE buffer. DNA concentration was determined by Qubit ^®^2.0 fluorometer (Life Technologies).

Bisulfite converted DNA and mRNA library preparation was performed in Georgia Genomics Facility (http://dna.uga.edu), and sequencing was performed using the Illumina NextSeq500 platform. These methods allowed for the detection of methylation at cytosine nucleotides (5mC) but did not allow for the detection of 5-hydroxymethylcytosine (5hmC)^58^. Raw RNA-seq and BS-seq reads were trimmed for quality and adapter contamination using Trimmomatic^59^. All sequence data related to this project have been deposited in NCBI’s SRA; BioProject ID: PRJNA309979.

### Analysis of gene expression

Tophat2 (v2.0.12, ref. 60) was used to map strand-specific RNA-seq reads to the *Z. nevadensis* genome (v1.0; ref. 21). FPKM (fragments per kilobase of exon per million fragments mapped) produced by Cuffnorm^61^ was used to quantify expression levels of genes. Read counts for each locus were also established using the htseq-count script of the DESeq2 package^62^, and utilized for differential expression testing.

Cufflinks^61^ was used to generate library-specific transcriptome annotations, which were then merged using cuffmerge. This merged cufflinks annotation was then resolved with the OGS annotations, and any multi-exon cufflinks transcript that did not overlap an OGS gene model was kept.

DESeq2^62^ was used to assess differential expression at all annotated loci, using mapped read counts provided by htseq-count. Caste and sex were modeled as independent variables, allowing for the testing of each while controlling for the other, utilizing a likelihood ratio test. Only genes with at least 1 read in all samples were kept for testing. As for differential methylation, we also performed differential expression tests between each relevant caste or sex pair, which were further combined to establish genes consistently differentially expressed between both caste or sex pairs.

We also performed a separate analysis of differential expression incorporating two previously published soldier caste samples with equivalent replication across both sexes (3 male, 3 female; ref. 21). We compared expression in each of the three castes (alate, worker, soldier) to the remaining two (while controlling for sex) to identify putatively up- and down-regulated genes. We also performed a second test for differential expression between sexes (after controlling for caste) utilizing the information from all three castes.

We established a measure of antisense transcription from our RNA-seq data. We first quantified the number of reads mapping to the sense and anti-sense direction of all gene models that did not overlap >50% of another gene mode. We then utilized a binomial-test to identify genes that were significantly transcribed from the antisense strand^63^. We then used a binomial test to assess the probability that a given locus in a given sample exhibited antisense transcription no different from that observed across all loci. Each locus within each library was judged as possessing significant antisense transcription based upon an FDR-corrected binomial p-value <0.05. Finally, we designated a locus as significantly expressed in antisense if >1/3^rd^ of libraries exhibited significant antisense transcription. We also produced a continuous metric of putative antisense transcription level for each sample type by averaging the proportion of all reads that map to the antisense strand of a given gene across all three replicates of each sample type.

DEXSeq^64^ was used at assess differential expression of exons independent of differences in gene expression across all multi-exon genes, after filtering out genes without read coverage in any sample. We tested both caste and sex in a combined model framework in order to identify caste- and sex-specific differences in exon inclusion.

### Analysis of DNA methylation

Bisulfite-converted reads obtained from pooled individuals were mapped to the *Z. nevadensis* genome using Bismark (v0.14.4; ref. 65), followed by duplicate removal. Reads were then used to infer methylation levels of cytosines genome-wide using a binomial test. This test incorporated deamination rate (from an unmethylated control) as the probability of success, and assigned a significance value to each CpG site related to the number of unconverted reads (putatively methylated Cs) as they compared to the expected number from our unmethylated lambda spike-in control. Resulting *P*-values were then adjusted for multiple testing^66^. Only sites with false discovery rate (FDR) corrected binomial *P* values <0.01 with more than three reads were considered “methylated”. Fractional methylation values were calculated as described previously^12^,^36^ for each CpG site or for each genomic feature (exons and introns). We observed no difference in the bisulfite conversion efficiency between libraries, as determined by analysis of a spike-in unmethylated lambda genome, as well as by assessment of methylation rate at non-genic non-CpG cytosines (Table S2). General metrics of DNA methylation were derived from a combined dataset produced by pooling all samples, unless otherwise indicated.

Methylation data from *A. mellifera* and *C. floridanus*, as well as *Z. nevadensis*, were obtained by trimming sequencing reads (GSM497246, GSE31577) and then mapping these read to their respective genomes (Amel 4.5, Cflo v3.3) using Bismark. CpG DNA methylation was then quantified and associated with features using the same methods as for *Z. nevadensis*. For *C. floridanus*, we only utilized a subset of samples (queen, worker, and males) in order to ensure similar BS-seq coverage between species. Species-level comparisons of DNA methylation were performed with datasets produced by pooling all considered samples within species. Orthologous relationships between genes were then established using Orthodb^67^ for all genes with 0 or 1 copy in any species.

Methylsig^68^ was used to assess differential methylation between samples. We assessed whether DNA methylation differed significantly between castes or sexes using 200 bp windows as established within the Methylsig pipeline (methylSigTile function). We also performed tests at the single-CpG level in order to produce a list of differentially methylated cytosines. We combined replicates of both sexes together for each caste (4 samples for each caste; 2 male and 2 female) when testing for differences in methylation between castes. Similarly we combined replicates of both castes together for each sex (4 samples for each caste; 2 worker and 2 alate) when testing for differences between sexes. For these tests, we required that at least 3 replicates of each caste or sex possess >4 reads at tested CpGs/windows and be methylated in at least half of the samples in order for a CpG or window to be tested. For both DMR and DMC testing using MethylSig, we utilized dispersion information from 200 bp flanking the DMR/DMC to bolster dispersion estimation (local.disp = T,winsize.disp = 200). Ajacent DMRs showing significant hypermethylation in the same sample type were merged. This resulted in a total of 862,025 CpGs (among 179,955 windows) with sufficient coverage and methylation status.

We also performed Methylsig analysis for all relevant caste and sex pairs (AF vs WF + AM vs WM and AF vs AM + WF vs WM, respectively) to produce a more conservative list of caste- and sex-associated DMR-containing genes that significantly differed between both pairs of a given comparison. Differentially methylated regions were then intersected with genic elements from the *Z. nevadensis* official gene annotation (v2.2), and each DMR was assigned the genic feature it overlapped most. Only DMRs exhibiting >15% absolute methylation difference and a FDR of <0.05 were utilized to establish differentially methylated genes.

We compared the number of DMRs or DMCs obtained by methylsig to the number of produced when shuffling replicates between comparisons (Fig. S8, top two panels). This analysis provided an estimate of potential false positives in our data. We also performed a second assessment of false discovery in our data using t-tests, which detected the presence of differential DNA methylation, after shuffling replicates between treatments at each window, for all testable windows of 200 bp. We then assessed the number of windows showing significant differential methylation after correcting for false discovery. We performed this analysis 100 times and then compared the number of observed DMRs among these 100 permutation tests to the number observed when comparing caste and sex using the same method (Fig. S8, bottom panel).

### Gene Orthology

We utilized Orthodb ortholog relationships for all ortholog groups with 1-to-1 representation in *A. mellifera, C. floridanus*, and *Z. nevadensis* (4,779 orthologs). In order to quantify large-scale patterns of gene gain and loss we also utilized orthodb^67^ gene families from across all insect species. We calculated the average proportion of species with a member ortholog (large-scale conservation), average copy number of the given ortholog group across species (ancestral duplication rate), and the ratio of *Z. nevadensis* copy number to average cross-insect copy number (ancestral-normalized *Z. nevadensis* duplication rate) for each gene family with representation in *Z. nevadensis*. This allowed for the estimation of large-scale evolutionary patterns for each ortholog group, in lieu of alternative evolutionary metrics that are complicated by the absence of a closely related species with genome data.

### MOTIF Analysis

We developed DMR test sets by extracting 150 bp of genomic sequence surrounding confidently differentially methylated cytosines (FDR < 0.01, absolute methylation change >20% between castes or sexes). Our control sets were constructed in the same manner, but were constructed around non-significantly differentially methylated cytosines (methylation level >30%) falling within 1.5 kb up- or down-stream of (but not overlapping) tested DMCs in order to account for variation in CpG content among the focal regions. This produced approximately 2-4x the number of control sequences for each test set.

We performed transcription factor binding site (TFBS) motif enrichment tests with the program Clover^69^. We compared enrichment of each TFBS profile within our test sequences relative to both control sequences used above, as well as all methylated introns. We also performed subsequent tests with AME^70^ to complement our Clover-based TFBS analysis using known TF binding motifs (flyreg^31^; idmmpmm^32^). We considered a TFBS motif enriched within DMCs if both tests (AME and clover) showed the TFBS was significantly enriched. Finally, we evaluated whether each significantly enriched TFBS motif was enriched within caste- or sex-specific DMC test sequences relative to the alternative’s test sequences (e.g., for caste DMC-surrounding sequences, we used sex DMC-surrounding sequences as a control) to isolate highly-confident TFBSs enriched within phenotype-specific DMCs.

We also used AME^70^ to detect *D. melanogaster* miRNA motifs over-represented in differentially methylated regions of the *Z. nevadensis* genome (miRbase-dme^71^). In order to roughly quantify the fold-overrepresentation of a given miRNA within our test sequences (relative to control), we used the FIMO program^72^ to scan our test and control sequences for the given miRNA profile. We then compared the test set size-normalized counts of significant hits (FDR < 0.1) between test and control sequences.

We investigated *de novo* motif enrichment (Table S14) with the program MEME^73^, (with the options -minw 6 -maxw 25 -revcomp -mod zoops) using DMC-surrounding sequences. We then used TOMTOM to compare enriched motifs to known motif databases.

### Data Availability

All sequence data related to this project have been deposited in NCBI’s SRA; BioProject ID: PRJNA309979.

## Additional Information

**How to cite this article**: Glastad, K. M. *et al*. The caste- and sex-specific DNA methylome of the termite *Zootermopsis nevadensis. Sci. Rep.* **6**, 37110; doi: 10.1038/srep37110 (2016).

**Publisher's note**: Springer Nature remains neutral with regard to jurisdictional claims in published maps and institutional affiliations.

## Supplementary Material

Supplementary Information

## Figures and Tables

**Table 1:** Numbers of differentially methylated genes (DMG) and differentially expressed genes (DEG) between termite castes, alate (A) or worker (W), and sexes, female (F) and male (M).

	Phenotype	Total	More highly methylated or expressed	
DMG	caste	2,720	A	W
			2,593	127
	sex	368	F	M
			192	176
DEG	caste	1,094	A	W
			599	495
	sex	834	F	M
			792	42

**Table 2:** Multiple transcription-factor-binding-site (TFBS) motifs are enriched within differentially methylated regions (DMRs).

DMR type	TFBS	FDR^a^	Comparison^b^	Znev gene ID	Name
Caste	Eip74EF	7.59E-06	caste	Znev_00833	Ecdysone-induced protein 74EF
	fkh	1.00E-05	caste	Znev_13477	fork head
	Ubx	3.93E-05	caste	Znev_15380	ultrabithorax
	bab1	7.15E-06	caste	Znev_03179	bric a brac
	tll	3.24E-03	ns	Znev_12982	tailless
	en	5.28E-03	caste	Znev_15553	engrailed
	srp	2.94E-02	ns	Znev_02318	serpent
Sex	SuH	7.34E-04	ns	Znev_04163	supressor of hairless
	nub	8.55E-03	sex	Znev_14256	nubbin
	vvl	1.15E-02	ns	Znev_11549	ventral veins lacking
	z	2.03E-02	sex	Znev_02821	zeste

Putative TFBSs that were significantly enriched within differentially methylated cytosine-centered sequences.

^a^The FDR-corrected p-value of motif enrichment analysis using the program AME.

^b^Value indicating if a given TFBS was enriched among either caste or sex DMRs when using the opposite as control sequences. ns: caste vs sex comparison nonsignificant.

**Table 3:** Differentially methylated regions (DMRs) are enriched for miRNA profiles.

DMRs	miRNA	Positive hits^a^	Negative hits^a^	Fold enrichment	AME FDR
Caste-biased	Zne-mir-34-3p	55	0	103.337	8.97E-13
	Zne-mir-263a-3p	24	0	45.092	1.11E-10
	Zne-mir-6012-5p	267	16	31.353	3.03E-15
	Zne-mir-2a-3-5p	10	0	18.788	1.39E-11
	Zne-mir-125-5p	10	0	18.788	1.54E-02
	Zne-mir-2796-3p	5	0	9.394	4.38E-02
	Zne-mir-279c-5p	4	0	7.515	5.97E-10
	Zne-mir-3049-5p	4	0	7.515	4.41E-06
	Zne-mir-87-1-3p	14	4	6.576	1.43E-05
	Zne-mir-981-5p	3	0	5.637	1.57E-12
	*all miRNAs*^b^	556	136	7.681	
Sex-biased	Zne-mir-34-3p	19	0	35.005	4.68E-04
	Zne-mir-275-3p	9	0	16.581	4.13E-04
	Zne-mir-998-5p	7	0	12.897	6.44E-03
	Zne-mir-750-5p	6	0	11.054	3.80E-02
	Zne-bantam-5p	14	4	6.448	3.72E-02
	Zne-mir-6012-3p	3	0	5.527	1.58E-02
	Zne-mir-278-5p	3	0	5.527	1.79E-02
	Zne-mir-981-5p	2	0	3.685	1.09E-05
	Zne-mir-279c-5p	2	0	3.685	1.67E-02
	Zne-mir-184-5p	1	0	1.842	3.45E-05
	*all miRNAs*^b^	222	110	3.718	

Top 10 miRNA sequences showing overrepresentation among sequences surrounding caste- and sex-biased differentially methylated cytosines.

^a^The number of significant hits (FIMO) for the given miRNA among caste- and sex-biased DMRs (+hits) as well control sequences–N *positive set*, caste: 5,786, sex: 1,364; N *negative set*, caste: 10,871, sex: 2,513.

^b^The number of positive and negative set sequences featuring a significant hit to *any* miRNA.
