# Supplementary material for: The caste- and sex-specific DNA methylome of the termite Zootermopsis nevadensis
Source: Sci Rep. 2016 Nov 16;6:37110. doi: 10.1038/srep37110 (PMC5111047; doi:10.1038/srep37110)
Supplement: Supplementary Information [file srep37110-s1.pdf]

Supporting Information for **The caste- and sex-specific DNA methylome of the termite *Zootermopsis nevadensis***

Karl M. Glastad, Kaustubh Gokhale, Jüergen Liebig, and Michael A. D. Goodisman

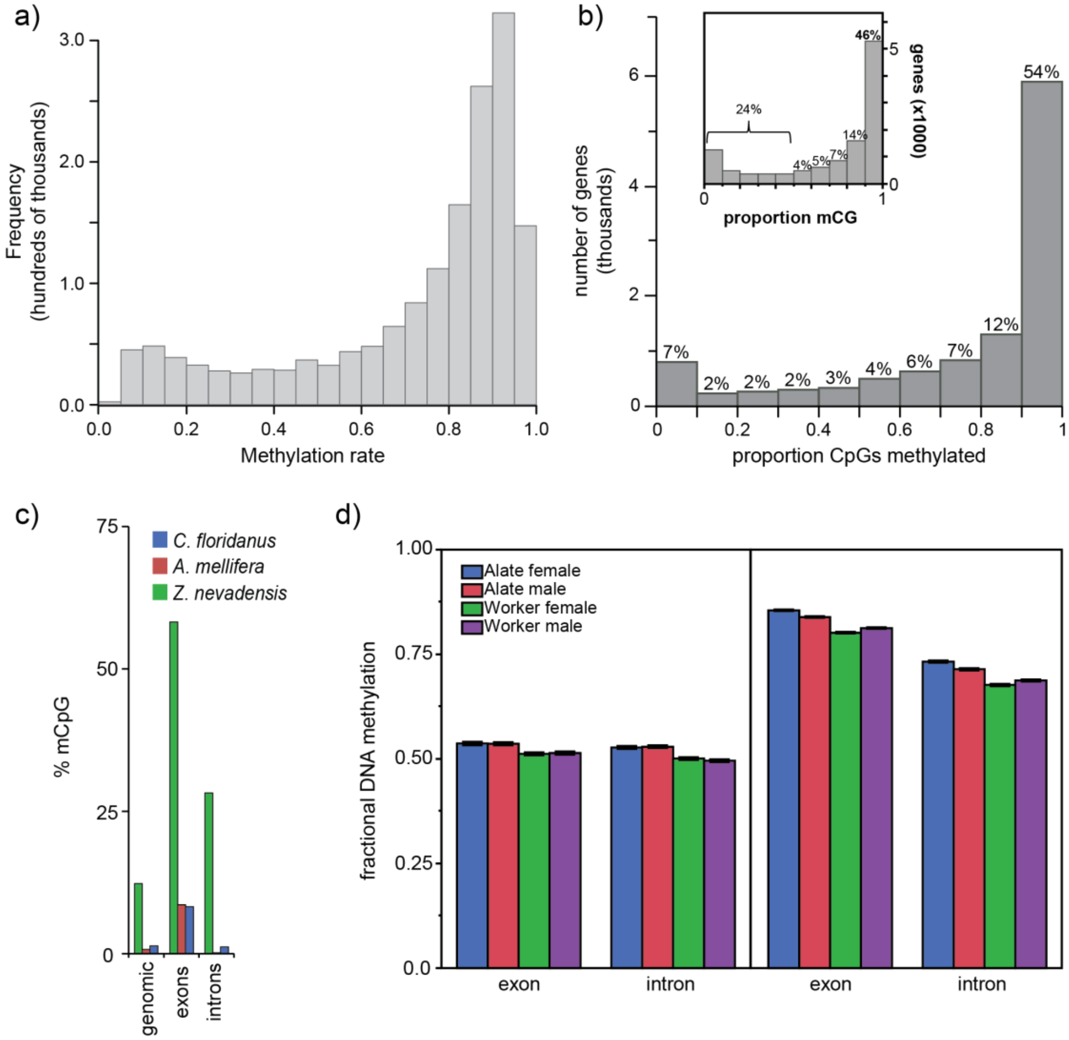

Fig. S1: **DNA methylome of *Z. nevadensis*.** (a) Methylation of methylated CGs in the *Z. nevadensis* genome showing that the majority of mCGs are highly methylated (>0.75). (b) Histogram of proportions of exonic CpGs that are methylated, showing >50% of methylated genes possess >=90% of CpGs that are methylated (inset: same, but for CpGs falling with exons and introns). (c) Percentage of methylated CpGs out of all CpGs genome-wide, within exons, and within introns for three insect species. (d) Fractional methylation level of alate females, alate males, worker females, and worker males for all exons and introns (left), as well as all exons and introns methylated in two or more phenotypes (right). Error bars: 95% confidence interval of mean.

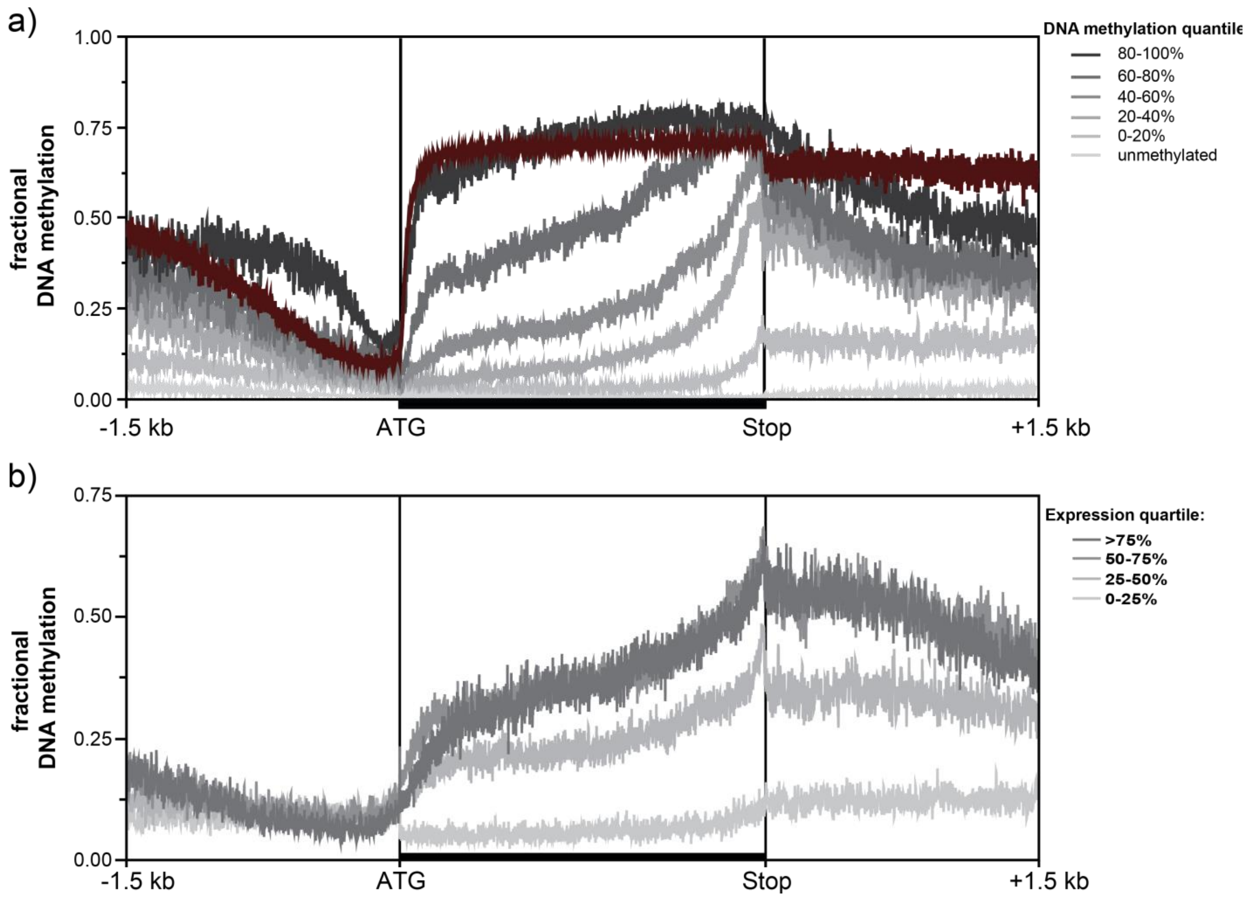

Fig. S2: Fractional methylation across termite genes. (a) Positional methylation levels for genes of differing methylation in *Z. nevadensis* genes (grey) and human genes (maroon). (b) Positional methylation levels for genes of increasing expression level quantiles in *Z. nevadensis*.

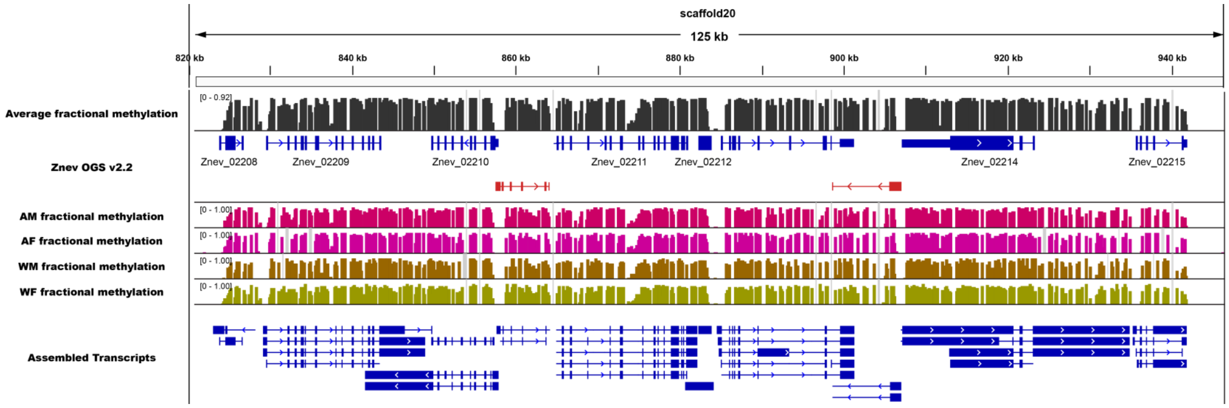

Fig. S3: Genome browser snapshot of a high-methylation region showing termite DNA methylation as it relates to known (blue) and novel (red) gene models across all four *Z. nevadensis* morphs.

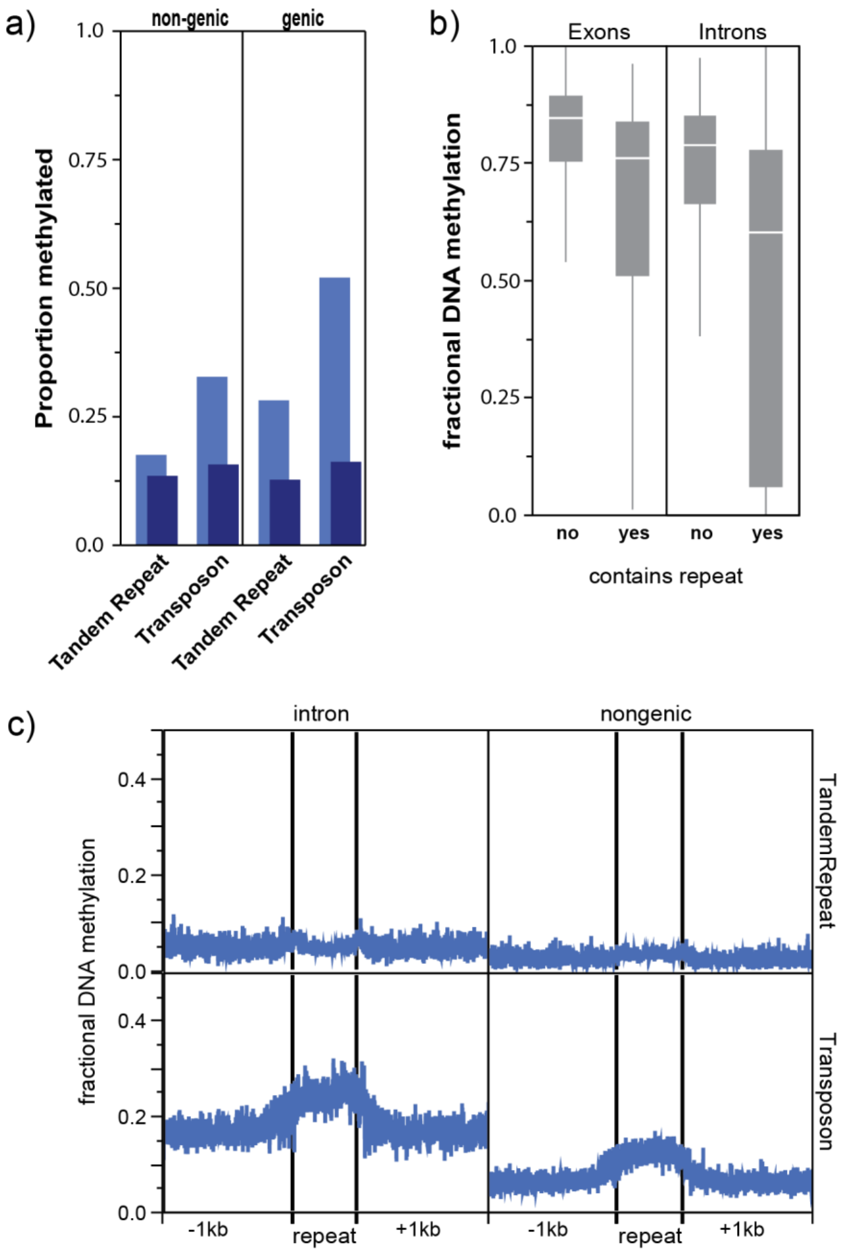

Fig. S4: DNA methylation at *Z. nevadensis* repeats. (a) Proportion of different repeat types showing evidence of DNA methylation (> 2 methylated CpGs) among repeats falling inside (genic) and outside (non-genic) of genes (light blue), as well as for repeats lacking DNA methylation within the surrounding 500bp up- and down-stream region (dark blue), (b) average methylation level of methylated exons and introns based upon whether they contain a repeat or not, and (c) spatial DNA methylation profiles within and surrounding repeats falling within introns, as well as those falling outside of genes (nongenic).

Term	*r*	partial
CV	-0.509	-0.247
expression difference	-0.394	-0.158
gene-gene distance	-0.276	-0.205
speceis duplication	-0.251	-0.057
expression level	0.420	0.061
conservation	0.249	0.054
antisense expression	0.251	0.198
exon count	0.413	0.263

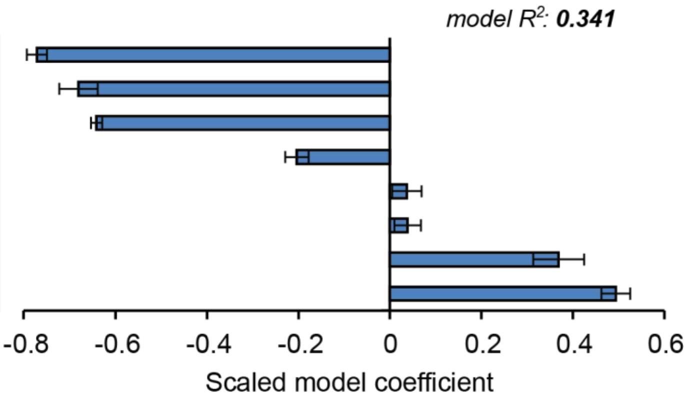

Fig. S5: **Multivariate analysis of major correlates of termite DNA methylation**. Pearson's correlation coefficients and partial correlation coefficients are given for correlations of eight variables with DNA methylation. Bar graph provides scaled model coefficients from a combined regression analysis of all eight variables against gene DNA methylation levels. Error bars: 95% confidence interval of the mean. CV: coefficient of variation.

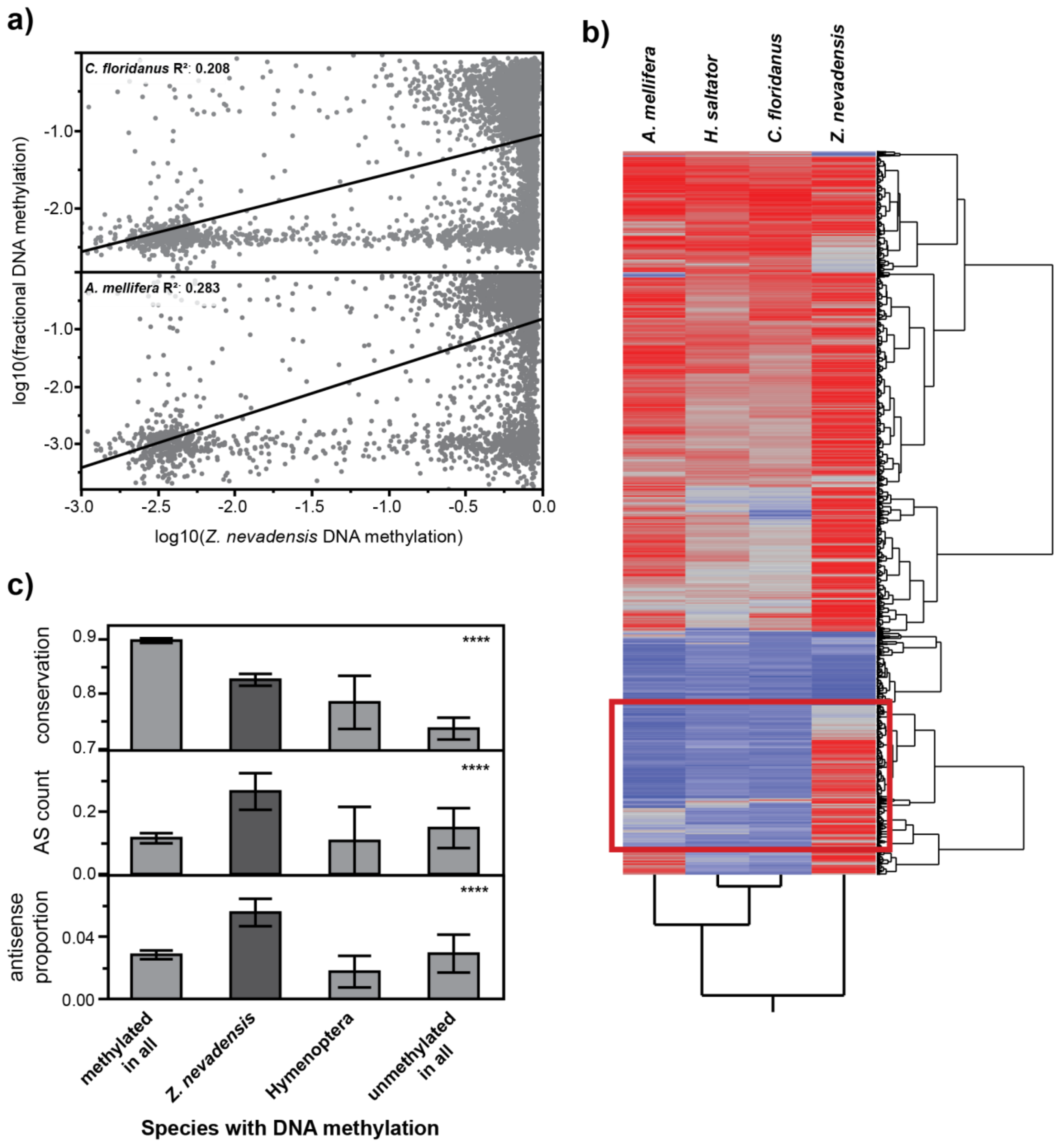

Fig. S6: DNA methylation in *Z. nevadensis* is expanded relative to methylation in the Hymenoptera. (a) Regression between *Z. nevadensis* DNA methylation and DNA methylation levels for orthologs in *C. floridanus* (top) and *A. mellifera* (bottom) (b) Hierarchical clustering (ward method) of ~5,000 orthologs between *Z. nevadensis* and hymenopteran social insects illustrating large class of genes with *Z. nevadensis*-specific methylation (red box), which (c) exhibit distinct qualities relative to methylated or unmethylated genes. Hymenoptera: genes methylated in ants and bees but not *Z. nevadensis*. Error bars: 95% confidence interval of mean.

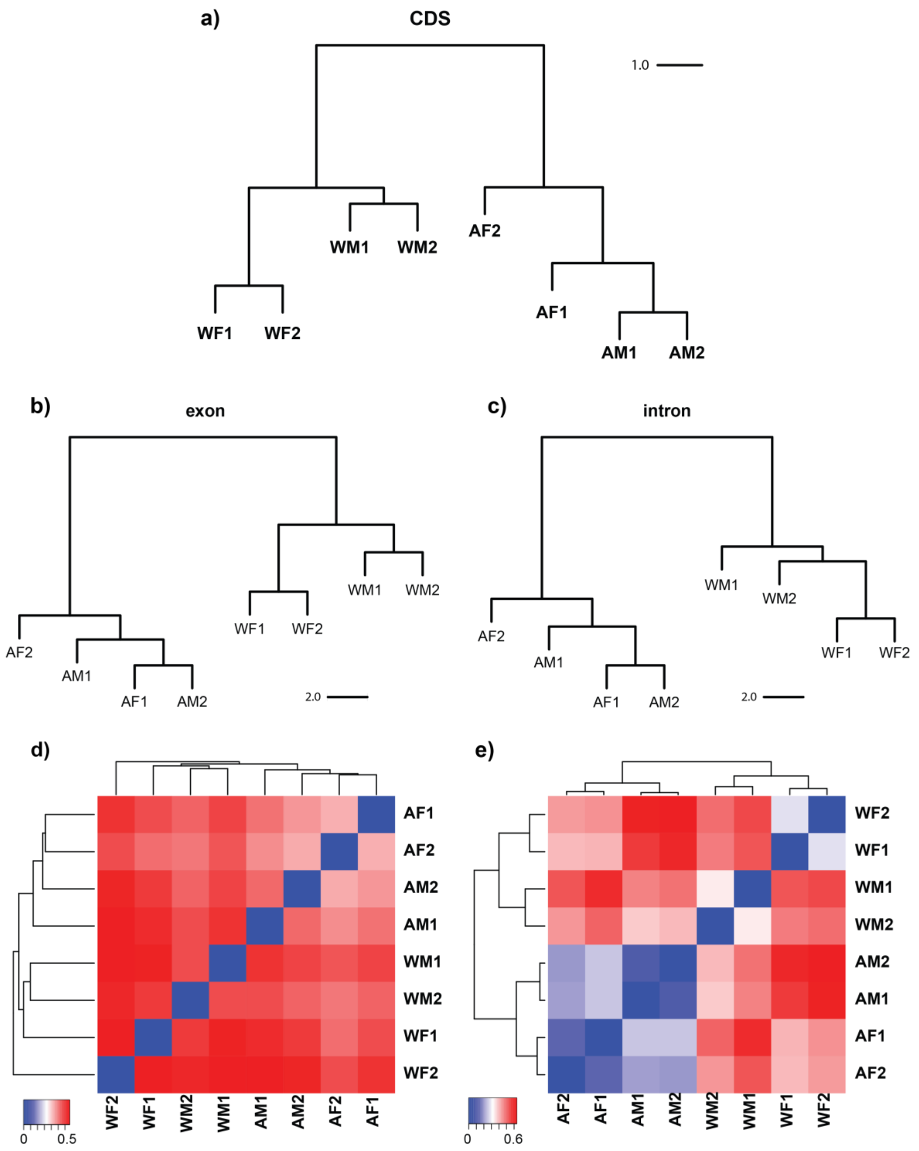

Fig. S7: **Dendrograms representing hierarchical clustering of DNA methylation libraries based upon DNA methylation levels.** Dendrograms considering information from within (a) coding sequences (CDS; combined exons), (b) exons, and (c) introns illustrating strong caste-based clustering of methylation libraries. (d) Methylation library heatmaps for all mCGs shared between four or more libraries or (e) all differentially methylated CpGs (DMCs).

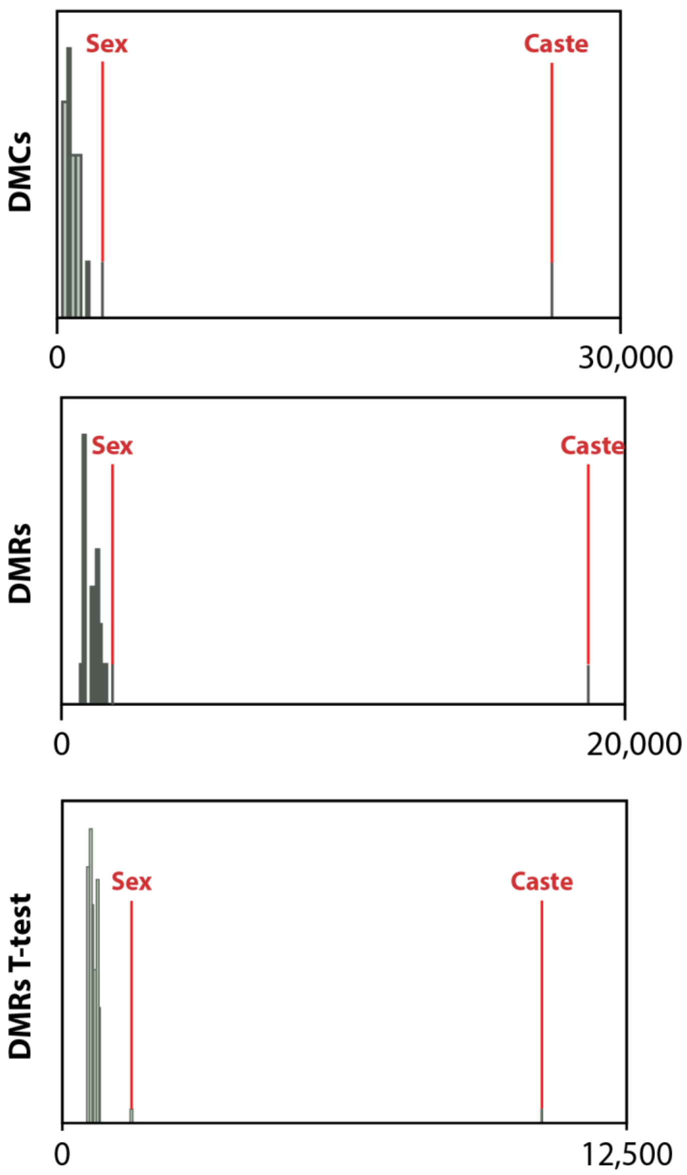

Figure S8: Histograms showing results from permutation tests for caste and sex DMCs (top row) and DMRs (middle row). The number of predicted significantly differing DMRs/DMCs is given for each set of replicate permutations (grey bars), along with the actual number of significantly differing DMRs/DMCs observed for the given comparison (indicated in red). Also included are the results of a separate set of permutation tests performed on 200bp windows utilizing t-tests (bottom row). For this analysis, observed numbers of significant (by t-tests) DMRs for comparisons between caste and sex (red) were compared to the number of significant DMRs observed when replicates were shuffled. For the expected distribution (shuffled replicates) replicates were re-shuffled for each tested window, and the total number of significantly differing DMRs were determined. This was performed 100 times, and the number of DMRs for these 100 permutations were compared to the number observed between caste and sex comparisons.

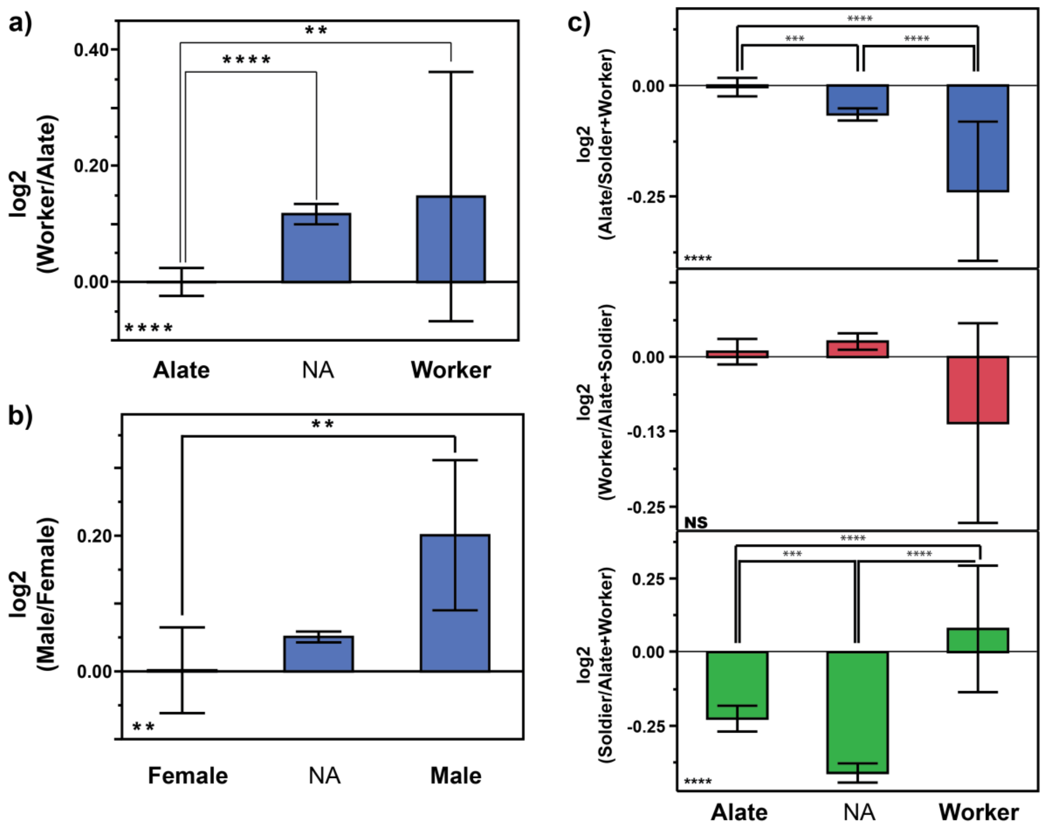

Fig. S9: **Differentially metylated genes (DMGs) are more highly expressed in more highly methylated phenotypes**. Ratio of gene expression by DMG up-methylation type for (a) caste expression ratio by caste biased DMG type and (b) sex expression ratio by sex biased DMG type. (c) Caste biased DMGs were also compared to ratios of gene expression showing single caste-specific expression for each of three castes: Alate (top), Worker (middle), and Soldier (bottom). Bottom left of each graph features Kruskal-Wallis significance test Pvalue. All other pavlues related to wilcoxon *post hoc* pairwise tests (given full test significance). Error bars: 95% confidence interval of mean.

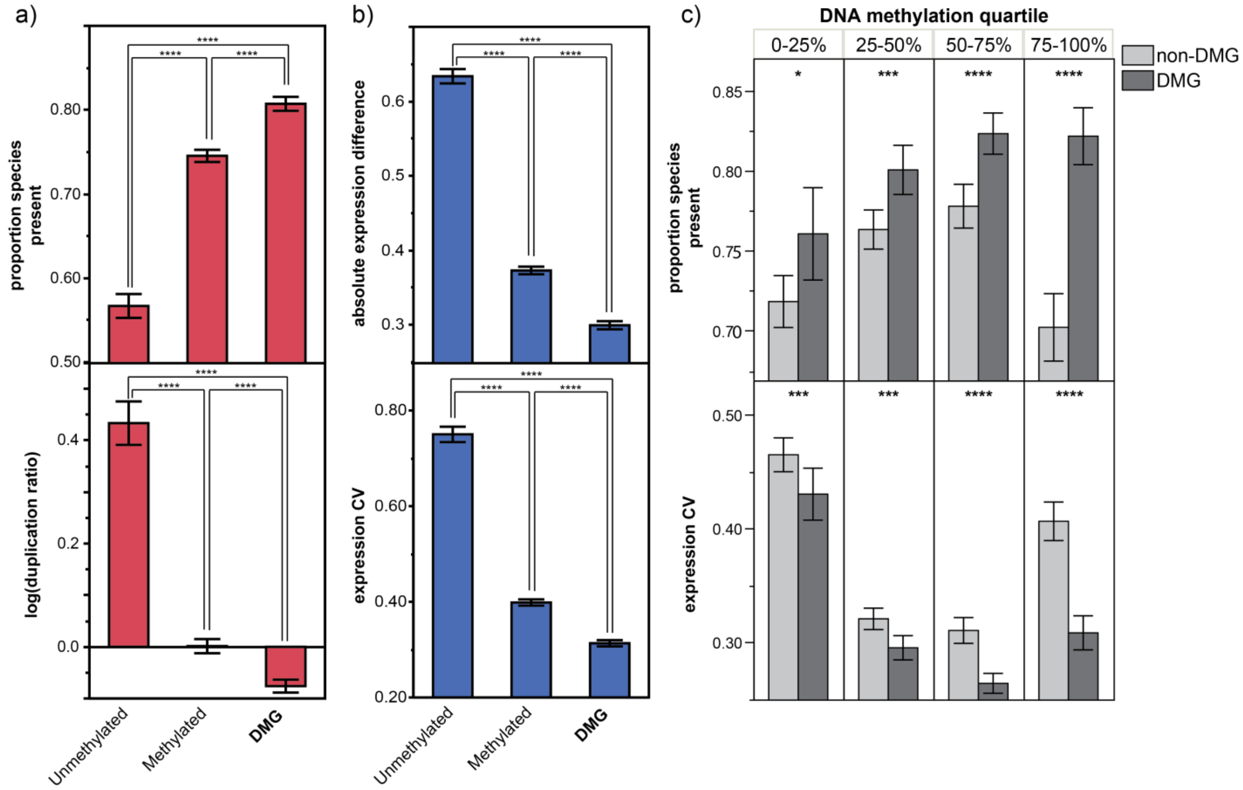

Fig. S10: Differentially methylated genes show distinct expression and evolutionary conservation. (a) Gene conservation (top) and *Z. nevadensis* duplication ratio (*Z. nevadensis* orthodb copy number/insect-wide orthodb copy number; bottom). (b) Absolute gene expression difference between four morphs (top) and gene expression variation (bottom) for unmethylated, methylated (but not differentially-methylated), and differentially methylated genes (DMG). (c) Expression coefficient of variation and gene conservation presented for differentially methylated genes and non-differentially methylated genes (non-DMG) across four quartiles of DNA methylation level, showing that DMGs differ from non-DMGs consistently across methylation levels. P-values from (a) and (b) from Wilcoxon *post hoc* pairwise tests (all comparisons significant at group level) and (c) Wilcoxon rank-sum tests. Error bars: 95% confidence interval of mean.

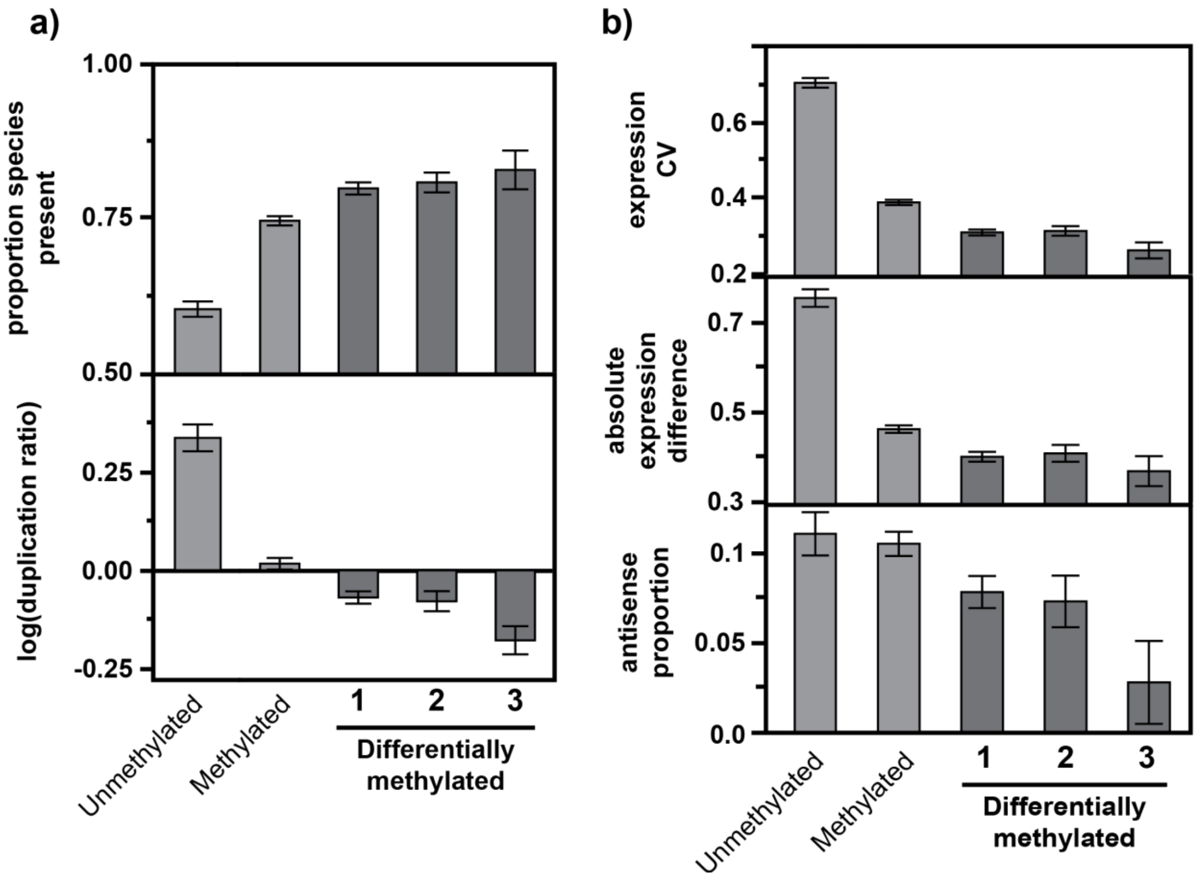

Fig. S11. Differentially methylated genes are more conserved and less variably expressed than unmethylated or non-differentially methylated genes. (a) Proportion of species with a representative Orthodb ortholog group member present (top), and *Z. nevadensis* gene copy number to average copy number across insect species (bottom) for unmethylated genes, methylated genes, and differentially methylated genes exhibiting differential methylation in one, two, or three or more (1-3 respectively) pairwise tests (of four possible tests). (b) gene expression coefficient of variation (top), absolute sample expression fold difference (middle), and proportion of gene reads that map to antisense strand (bottom) for different gene methylation classes. Error bars: 95% confidence interval of mean.

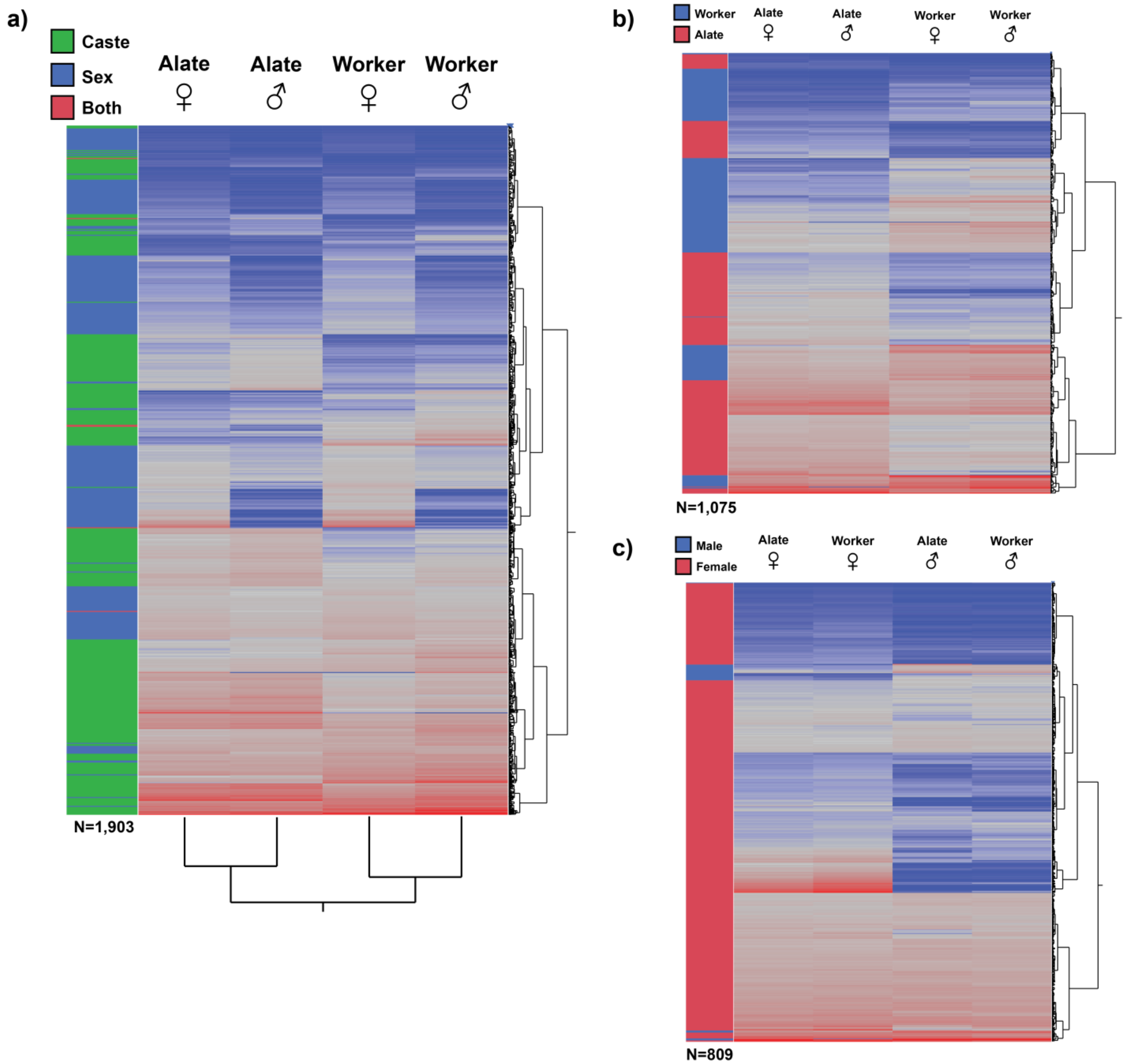

Fig. S12: Hierarchical clustering of genes showing evidence of differential expression between castes or sexes. (a) All genes showing differential expression between either castes or sexes. (b) Clustering of only genes differentially expressed between castes and (c) sexes.

Table S1: library read statistics for both RNA- and BS- sequencing libraries.

Library	Library	replicate	sample[a]	Raw reads	Mapped reads	Average coverage
dAF1	BS-seq	1	AF	69,857,248	39,750,815	13.32
dAF2	BS-seq	2	AF	53,229,272	27,257,321	8.75
dAM1	BS-seq	1	AM	121,948,184	58,316,642	17.49
dAM2	BS-seq	2	AM	104,010,010	59,634,522	19.89
dWF1	BS-seq	1	WF	128,978,050	57,231,091	15.55
dWF2	BS-seq	2	WF	116,144,222	56,091,051	16.83
dWM1	BS-seq	1	WM	65,700,056	32,320,740	9.70
dWM2	BS-seq	2	WM	77,363,986	35,689,828	10.71
rAF1	RNA-seq	1	AF	127,281,074	100,379,415	x
rAF2	RNA-seq	2	AF	52,923,776	25,655,228	x
rAF3	RNA-seq	3	AF	76,223,816	43,673,913	x
rAM1	RNA-seq	1	AM	90,723,714	64,866,998	x
rAM2	RNA-seq	2	AM	108,295,460	61,763,464	x
rAM3	RNA-seq	3	AM	93,942,542	48,922,444	x
rWF1	RNA-seq	1	WF	84,454,742	67,632,139	x
rWF2	RNA-seq	2	WF	99,997,290	69,612,820	x
rWF3	RNA-seq	3	WF	55,324,838	24,899,460	x
rWM1	RNA-seq	1	WM	94,984,334	68,974,616	x
rWM2	RNA-seq	2	WM	80,912,886	62,080,059	x
rWM3	RNA-seq	3	WM	79,851,626	45,633,726	x

[a]Sample IDs: AF, alate female; AM, alate male; WF, worker female; WM, worker male.

Table S2: Conversion levels for cytosines as a proportion of mapped reads within lambda control and genomic DNA for CG, CHG, and CHH nucleotides:

Sample[a]	CG genomic	CHG+CHH genomic	CpG lambda	CHH+CHG lambda
**AF1**	0.104	0.005	0.004	0.005
**AF2**	0.101	0.004	0.004	0.004
**AM1**	0.102	0.004	0.004	0.004
**AM2**	0.105	0.004	0.004	0.004
**WF1**	0.105	0.004	0.004	0.004
**WF2**	0.101	0.004	0.004	0.004
**WM1**	0.103	0.004	0.004	0.004
**WM2**	0.106	0.004	0.004	0.004

[a]Sample IDs: AF, alate female; AM, alate male; WF, worker female; WM, worker male.

Table S3: Level of genomic CpG methylation in Znev libraries. First five rows represent CpGs featuring greater than three reads in all sample types.

Library[a]	CGs[b]	mCGs[c]	Prop mCG[d]
AF	11,770,583	1,377,158	0.117
AM	11,770,583	1,400,699	0.119
WF	11,770,583	1,378,272	0.117
WM	11,770,583	1,353,617	0.115
total – union	11,770,583	1,471,312	0.125
total – intersection	11,770,583	1,345,230	0.114
Species-level	13,027,374	1,628,422	0.125

[a]AF, alate female; AM, alate male; WF, worker female; WM, worker male; total union: total number of CGs methylated in any of the four sample types; total – intersection: total number of CGs methylated in all sample types; species-level, statistics from all libraries merged into one and treated as a single sample.

[b]CGs represents total number of CpG's featuring >3 reads.

[c]binomial test-determined "methylated" CpGs;

[d]proportion of CpGs that are methylated.

Table S4. Proportion of methylated CpGs and total number of CpGs with data within genomic features for *Z. nevadensis*, *A. mellifera*, and *C. floridanus*.

	Z. nevadensis		*A. mellifera*		*C. floridanus*	
	**% mCG**	**total CGs**	**% mCG**	**total CGs**	**% mCG**	**total CGs**
genomic	12.33	13,027,374	0.78	9,424,047	1.38	10,295,696
gene	33.99	3,130,951	1.53	4,412,751	3.79	3,066,266
exons	58.23	595,501	8.15	748,622	8.26	1,118,715
introns	28.21	2,532,521	0.16	3,660,706	1.20	1,962,896

Table S5: Differential methylation between *Z. nevadensis* castes or sexes. Number of differentially-methylated genes and number of hypermethylated genes in either of two compared sample types.

	phenotype		total	hyper-caste[d]	
combined framework[a]	caste			A	W
			2,720	2,593	127
	sex			F	M
			368	192	176
pairwise tests[b]	caste			A	W
		AF.WF	5,005	4,827	178
		AM.WM	3,284	2,846	438
		Shared[c]	1,820	1,777	43
	sex			F	M
		AF.AM	1,253	941	312
		WF.WM	2,388	933	1,455
		Shared[c]	225	151	74

[a]represents numbers of differentially methylated genes between caste and sex while controlling for the alternative.

[b]number of differentially methylated genes when comparing each representative pair separately for caste and sex.

[c]number of differentially methylated genes that show consistent directional differential methylation between both pairs of a given comparison (caste or sex).

[d]phenotype of hypermethylation for the given comparison.

Table S6: Genic localization for differentially methylated regions (DMRs) and differentially methylated cytosines (DMCs) for caste- and sex-significant DMRs or DMCs.

	comparison	Feature[a]	proportion of DMRs[b]	DMR count	tested regions
DMRs	caste	intron	0.547	10,015	105,965
		exon	0.285	5,221	42,555
		5prox	0.048	884	10,344
		3prox	0.119	2,181	21,089
		total		18,301	179,955
	sex	intron	0.569	799	105,965
		exon	0.239	336	42,555
		5prox	0.060	84	10,344
		3prox	0.132	186	21,089
		total		1,405	179,955
DMCs	caste	intron	0.499	13,425	450,657
		exon	0.334	8,999	271,357
		5prox	0.044	1,179	38,002
		3prox	0.123	3,304	102,007
		total		26,907	862,025
	sex	intron	0.510	1,017	450,657
		exon	0.295	589	271,357
		5prox	0.065	130	38,002
		3prox	0.130	260	102,007
		total		1,996	862,025

[a]5prox, 1.5kb upstream of gene start; 3prox, 1.5kb downstream of gene stop.

[b]Proportion of all significant DMRs that fall within given context.

Table S7: Gene ontology enrichment for genes featuring significant DNA methylation or featuring no DNA methylation in the *Z. nevadensis* genome.

Term	Category[a]	FDR	Fold enrichment	GO-ID
Methylated genes				
ATP binding	F	9.88E-30	5.74	GO:0005524
protein phosphorylation	P	4.01E-07	2.63	GO:0006468
DNA repair	P	4.05E-07	13.91	GO:0006281
macromolecular complex subunit organization	P	8.63E-07	2.42	GO:0043933
zinc ion binding	F	9.35E-07	1.73	GO:0008270
microtubule organizing center	C	3.22E-06	21.87	GO:0005815
protein serine/threonine kinase activity	F	3.24E-06	2.86	GO:0004674
spliceosomal complex	C	2.21E-05	8.23	GO:0005681
nucleoplasm part	C	3.10E-05	4.72	GO:0044451
ATP-dependent helicase activity	F	3.31E-05	16.42	GO:0008026
translation factor activity, RNA binding	F	6.25E-05	10.33	GO:0008135
purine ribonucleoside triphosphate catabolic process	P	6.25E-05	10.33	GO:0009207
histone modification	P	9.97E-05	7.60	GO:0016570
RNA splicing, via transesterification reactions with bulged adenosine as nucleophile	P	1.14E-04	4.34	GO:0000377
single-organism carbohydrate metabolic process	P	1.14E-04	4.37	GO:0044723
structural constituent of ribosome	F	2.33E-04	5.12	GO:0003735
spindle	C	3.65E-04	13.11	GO:0005819
chromosome	C	3.94E-04	2.47	GO:0005694
Golgi vesicle transport	P	5.43E-04	12.64	GO:0048193
oxidoreductase activity, acting on the CH-CH group of donors	F	8.18E-04	12.16	GO:0016627
oogenesis	P	8.98E-04	4.70	GO:0048477
nuclear envelope	C	1.25E-03	11.69	GO:0005635
motor activity	F	1.27E-03	8.32	GO:0003774
regulation of hydrolase activity	P	1.75E-03	4.46	GO:0051336

transferase activity, transferring acyl groups other than amino-acyl groups	F	2.23E-03	3.44	GO:0016747
mitotic nuclear division	P	2.43E-03	5.94	GO:0007067
microtubule-based movement	P	2.52E-03	13.34	GO:0007018
tRNA aminoacylation for protein translation	P	2.70E-03	10.52	GO:0006418
mitotic M phase	P	3.72E-03	12.87	GO:0000087
GTPase regulator activity	F	3.82E-03	7.61	GO:0030695
isomerase activity	F	4.18E-03	4.80	GO:0016853
ubiquitin-like protein transferase activity	F	4.18E-03	4.80	GO:0019787
regulation of Rho protein signal transduction	P	4.71E-03	4.17	GO:0035023
ribosomal subunit	C	4.84E-03	5.54	GO:0044391
monocarboxylic acid metabolic process	P	5.11E-03	5.70	GO:0032787
microtubule cytoskeleton organization	P	5.38E-03	2.91	GO:0000226
male gamete generation	P	5.40E-03	12.39	GO:0048232
endoplasmic reticulum membrane	C	5.40E-03	12.39	GO:0005789
microtubule associated complex	C	6.08E-03	9.58	GO:0005875
aminoacyl-tRNA ligase activity	F	6.34E-03	9.81	GO:0004812

Unmethylated genes

structural constituent of cuticle	F	5.56E-25	20.52	GO:0042302
sequence-specific DNA binding	F	5.30E-11	3.18	GO:0043565
odorant binding	F	8.27E-10	27.54	GO:0005549
sequence-specific DNA binding transcription factor activity	F	2.74E-08	2.35	GO:0003700
chitin binding	F	1.87E-07	5.89	GO:0008061
heme binding	F	3.27E-06	3.08	GO:0020037
neuropeptide receptor activity	F	4.13E-06	18.78	GO:0008188
oxidoreductase activity, acting on paired donors, with incorporation or	F	5.03E-06	3.01	GO:0016705

reduction of molecular oxygen				
chitin metabolic process	P	7.19E-06	4.21	GO:0006030
development of primary sexual characteristics	P	1.55E-05	3.83	GO:0045137
electron carrier activity	F	1.72E-04	2.62	GO:0009055
nucleosome	C	2.89E-04	5.91	GO:0000786
hormone activity	F	4.15E-04	9.52	GO:0005179
regulation of transcription, DNA-templated	P	4.85E-04	1.53	GO:0006355
integral component of membrane	C	6.85E-04	1.49	GO:0016021
extracellular region	C	7.18E-04	2.12	GO:0005576
nucleosome assembly	P	2.17E-03	4.33	GO:0006334
flavin adenine dinucleotide binding	F	2.41E-03	3.08	GO:0050660
cell fate specification	P	3.59E-03	3.39	GO:0001708
cullin-RING ubiquitin ligase complex	C	5.85E-03	2.89	GO:0031461
G-protein coupled amine receptor activity	F	6.23E-03	8.64	GO:0008227
carboxylic ester hydrolase activity	F	1.09E-02	3.20	GO:0052689
G-protein coupled receptor signaling pathway, coupled to cyclic nucleotide second messenger	P	1.25E-02	6.91	GO:0007187
positive regulation of sodium ion transport	P	1.80E-02	21.57	GO:0010765
Wnt signaling pathway, calcium modulating pathway	P	1.92E-02	7.56	GO:0007223
metalloexopeptidase activity	F	2.09E-02	3.96	GO:0008235
DNA integration	P	2.20E-02	5.76	GO:0015074
sodium channel activity	F	2.73E-02	3.46	GO:0005272
central nervous system development	P	3.33E-02	1.83	GO:0007417
neural tube development	P	3.54E-02	6.05	GO:0021915
axon extension	P	3.68E-02	4.94	GO:0048675
extracellular-glutamate-gated ion channel activity	F	4.42E-02	10.79	GO:0005234
enteroendocrine cell differentiation	P	4.42E-02	10.79	GO:0035883
cGMP biosynthetic process	P	4.42E-02	10.79	GO:0006182
regulation of muscle organ development	P	4.42E-02	10.79	GO:0048634
guanylate cyclase activity	F	4.42E-02	10.79	GO:0004383

specification of segmental identity, head	P	4.42E-02	10.79	GO:0007380
endocrine pancreas development	P	4.42E-02	10.79	GO:0031018
morphogenesis of a branching epithelium	P	4.62E-02	2.54	GO:0061138
negative regulation of multicellular organismal process	P	4.79E-02	3.60	GO:0051241

[a] P, biological process; F, molecular function; C, cellular component

Table S8: Gene Ontology terms associated with the highest and lowest two methylation deciles, relative to all other methylated genes.

Term	Category[a]	FDR	fold enrichment	GO-ID
Highest deciles				
chromatin modification	P	2.43E-03	3.22	GO:0016568
nucleic acid binding	F	2.73E-03	1.67	GO:0003676
transition metal ion binding	F	3.85E-03	1.67	GO:0046914
chromatin organization	P	7.88E-03	2.68	GO:0006325
chromosome organization	P	1.54E-02	2.19	GO:0051276
metal ion binding	F	1.54E-02	1.52	GO:0046872
macromolecule methylation	P	1.54E-02	4.55	GO:0043414
histone modification	P	1.54E-02	3.07	GO:0016570
covalent chromatin modification	P	1.54E-02	3.07	GO:0016569
zinc ion binding	F	1.54E-02	1.65	GO:0008270
nucleic acid metabolic process	P	1.59E-02	1.52	GO:0090304
methylation	P	1.59E-02	4.11	GO:0032259
nucleus	C	1.65E-02	1.59	GO:0005634
chromosome	C	1.94E-02	2.22	GO:0005694
macromolecular complex subunit organization	P	2.51E-02	1.88	GO:0043933
protein-lysine N-methyltransferase activity	F	2.51E-02	6.66	GO:0016279
lysine N-methyltransferase activity	F	2.51E-02	6.66	GO:0016278
S-adenosylmethionine-dependent methyltransferase activity	F	2.51E-02	4.05	GO:0008757
regulation of gene expression	P	3.67E-02	1.68	GO:0010468
transferase activity, transferring one-carbon groups	F	4.23E-02	2.56	GO:0016741
histone methylation	P	4.23E-02	5.18	GO:0016571
cell fate commitment	P	4.31E-02	2.92	GO:0045165
cation binding	F	4.62E-02	1.44	GO:0043169
multi-organism process	P	4.98E-02	1.79	GO:0051704
DNA methylation or demethylation	P	4.98E-02	41.20	GO:0044728
Lowest deciles				
signaling receptor activity	F	2.67E-05	3.28	GO:0038023
G-protein coupled receptor activity	F	2.73E-05	4.92	GO:0004930
receptor activity	F	2.73E-05	2.93	GO:0004872
transmembrane signaling receptor activity	F	8.47E-05	3.35	GO:0004888
integral component of membrane	C	9.14E-05	1.86	GO:0016021

intrinsic component of membrane	C	1.88E-04	1.68	GO:0031224
membrane	C	3.95E-04	1.47	GO:0016020
amino acid transmembrane transporter activity	F	1.26E-03	15.06	GO:0015171
heme binding	F	3.02E-03	3.54	GO:0020037
membrane part	C	3.22E-03	1.50	GO:0044425
molecular transducer activity	F	3.52E-03	1.90	GO:0060089
tetrapyrrole binding	F	3.52E-03	3.45	GO:0046906
transporter activity	F	3.59E-03	1.70	GO:0005215
signal transducer activity	F	3.81E-03	1.96	GO:0004871
carboxylic acid transmembrane transporter activity	F	4.72E-03	7.44	GO:0046943
organic anion transmembrane transporter activity	F	4.72E-03	7.44	GO:0008514
organic acid transmembrane transporter activity	F	4.72E-03	7.44	GO:0005342
transmembrane transporter activity	F	8.02E-03	1.71	GO:0022857
G-protein coupled receptor signaling pathway	P	9.91E-03	2.87	GO:0007186
circadian behavior	P	1.03E-02	20.03	GO:0048512
electron carrier activity	F	1.92E-02	2.99	GO:0009055
Wnt signaling pathway, calcium modulating pathway	P	2.13E-02	33.23	GO:0007223
circadian sleep/wake cycle	P	4.14E-02	16.67	GO:0042745
transmembrane transport	P	4.88E-02	1.73	GO:0055085
oxidoreductase activity, acting on paired donors, with incorporation or reduction of molecular oxygen	F	6.66E-02	2.63	GO:0016705

[a] P, biological process; F, molecular function; C, cellular component

TABLE S9 Terms associated with genes methylated in *Z. nevadensis* that are not methylated in *A. mellifera* and *C. floridanus.*

Term	Category[a]	FDR	fold enrichment	GO-ID
calcium ion binding	F	8.65E-07	4.29	GO:0005509
sequence-specific DNA binding	F	3.11E-06	4.82	GO:0043565
integral component of membrane	C	3.48E-05	2.15	GO:0016021
cell projection	C	4.59E-05	3.07	GO:0042995
extracellular region	C	2.10E-04	4.02	GO:0005576
nucleic acid binding transcription factor activity	F	3.04E-04	1.99	GO:0001071
G-protein coupled receptor signaling pathway	P	3.74E-04	6.57	GO:0007186
rhythmic process	P	5.73E-04	13.55	GO:0048511
open tracheal system development	P	6.05E-04	5.12	GO:0007424
eye development	P	6.05E-04	2.75	GO:0001654
locomotory behavior	P	2.15E-03	4.49	GO:0007626
epithelial cell migration	P	2.15E-03	5.75	GO:0010631
serine-type endopeptidase activity	F	3.08E-03	6.01	GO:0004252
transmembrane signaling receptor activity	F	3.09E-03	4.55	GO:0004888
membrane	C	4.81E-03	1.34	GO:0016020
proteinaceous extracellular matrix	C	4.81E-03	11.09	GO:0005578
sensory perception of chemical stimulus	P	5.46E-03	14.79	GO:0007606
axon choice point recognition	P	5.46E-03	14.79	GO:0016198
multi-organism behavior	P	7.10E-03	4.27	GO:0051705
sexual reproduction	P	9.04E-03	1.95	GO:0019953
cell fate commitment	P	1.16E-02	3.49	GO:0045165
homophilic cell adhesion via plasma membrane adhesion molecules	P	1.42E-02	9.86	GO:0007156
growth factor activity	F	1.68E-02	22.16	GO:0008083
formation of primary germ layer	P	1.79E-02	12.94	GO:0001704
imaginal disc pattern formation	P	2.02E-02	5.08	GO:0007447
multicellular organismal reproductive process	P	2.25E-02	1.87	GO:0048609
alpha-amino acid metabolic process	P	2.37E-02	2.62	GO:1901605
single organism reproductive process	P	2.64E-02	1.85	GO:0044702
synapse	C	2.88E-02	5.28	GO:0045202
regulation of transcription, DNA-templated	P	2.92E-02	1.54	GO:0006355
sensory organ morphogenesis	P	3.23E-02	2.29	GO:0090596
cell periphery	C	3.31E-02	2.03	GO:0071944
adult behavior	P	3.41E-02	4.03	GO:0030534
peptidase inhibitor activity	F	3.77E-02	5.55	GO:0030414
dorsal/ventral pattern formation	P	3.77E-02	5.55	GO:0009953

single organismal cell-cell adhesion	P	3.77E-02	5.55	GO:0016337
imaginal disc morphogenesis	P	3.77E-02	2.24	GO:0007560
cell-cell signaling	P	3.92E-02	2.50	GO:0007267
regulation of cell morphogenesis	P	4.41E-02	4.62	GO:0022604
plasma membrane part	C	4.70E-02	2.18	GO:0044459
enzyme linked receptor protein signaling pathway	P	4.70E-02	2.27	GO:0007167
protein phosphorylation	P	4.72E-02	1.69	GO:0006468
photoreceptor cell development	P	4.72E-02	3.43	GO:0042461
epithelial structure maintenance	P	4.72E-02	11.09	GO:0010669
serine-type endopeptidase inhibitor activity	F	4.72E-02	11.09	GO:0004867
mesoderm morphogenesis	P	4.72E-02	11.09	GO:0048332
response to wounding	P	4.72E-02	11.09	GO:0009611
embryonic heart tube morphogenesis	P	4.72E-02	11.09	GO:0003143
regulation of multicellular organismal process	P	4.92E-02	2.20	GO:0051239

[a] P, biological process; F, molecular function; C, cellular component

Table S10. Terms significantly enriched among differentially methylated genes that are methylated in *Z. nevadensis* but not *C. floridanus* or *A. mellifera*, relative to differentially methylated genes that are methylated in all species.

Term	Category[a]	FDR	fold enrichment	GO-ID
multicellular organismal process	P	3.01E-04	1.89	GO:0032501
single-multicellular organism process	P	3.01E-04	1.94	GO:0044707
anatomical structure morphogenesis	P	3.71E-04	2.49	GO:0009653
single-organism developmental process	P	1.02E-03	1.85	GO:0044767
sensory perception	P	1.46E-03	37.79	GO:0007600
cellular developmental process	P	3.69E-03	2.19	GO:0048869
anatomical structure development	P	3.69E-03	1.84	GO:0048856
signal transducer activity	F	7.67E-03	3.82	GO:0004871
multicellular organismal development	P	7.74E-03	1.79	GO:0007275
system development	P	7.74E-03	1.92	GO:0048731
sequence-specific DNA binding	F	7.74E-03	7.20	GO:0043565
biological adhesion	P	9.38E-03	5.34	GO:0022610
organ morphogenesis	P	1.05E-02	2.92	GO:0009887
cell development	P	1.12E-02	2.44	GO:0048468
protein dimerization activity	F	1.38E-02	8.40	GO:0046983
molecular transducer activity	F	1.45E-02	3.08	GO:0060089
organ development	P	1.45E-02	2.10	GO:0048513
cell differentiation	P	1.63E-02	2.07	GO:0030154
regionalization	P	1.63E-02	3.76	GO:0003002
system process	P	1.79E-02	4.20	GO:0003008
tissue development	P	2.07E-02	2.32	GO:0009888
extracellular region	C	2.07E-02	7.00	GO:0005576
imaginal disc pattern formation	P	2.07E-02	11.20	GO:0007447
cell surface receptor signaling pathway	P	2.72E-02	2.56	GO:0007166
proteinaceous extracellular matrix	C	3.00E-02	5.88	GO:0005578
nucleic acid binding transcription factor activity	F	3.07E-02	2.35	GO:0001071
signaling	P	3.28E-02	1.62	GO:0023052
calcium ion binding	F	3.44E-02	3.92	GO:0005509
neurological system process	P	3.44E-02	4.20	GO:0050877
respiratory system development	P	3.44E-02	5.13	GO:0060541
pattern specification process	P	3.44E-02	2.95	GO:0007389
extracellular region part	C	3.75E-02	8.40	GO:0044421
cellular component morphogenesis	P	4.28E-02	2.67	GO:0032989
epithelium development	P	4.55E-02	2.32	GO:0060429

[a] P, biological process; F, molecular function; C, cellular component

Table S11. Functional terms enriched for genes containing differentially methylated regions (DMGs) relative to methylated genes that do not contain any significantly-differing methylated regions.

Term	Category[a]	FDR	fold enrichment	GO-ID
protein binding	F	5.04E-10	1.30	GO:0005515
ATP binding	F	1.51E-07	1.62	GO:0005524
binding	F	9.33E-07	1.11	GO:0005488
adenyl ribonucleotide binding	F	1.28E-06	1.50	GO:0032559
adenyl nucleotide binding	F	1.28E-06	1.50	GO:0030554
nucleoside phosphate binding	F	1.28E-06	1.40	GO:1901265
nucleotide binding	F	1.28E-06	1.40	GO:0000166
purine nucleoside binding	F	1.28E-06	1.47	GO:0001883
purine ribonucleoside binding	F	1.28E-06	1.47	GO:0032550
ribonucleoside binding	F	1.28E-06	1.47	GO:0032549
purine ribonucleoside triphosphate binding	F	1.28E-06	1.47	GO:0035639
nucleoside binding	F	1.35E-06	1.47	GO:0001882
small molecule binding	F	2.37E-06	1.38	GO:0036094
anion binding	F	2.56E-06	1.41	GO:0043168
purine ribonucleotide binding	F	4.25E-06	1.41	GO:0032555
ribonucleotide binding	F	4.25E-06	1.41	GO:0032553
purine nucleotide binding	F	4.25E-06	1.41	GO:0017076
carbohydrate derivative binding	F	1.04E-05	1.38	GO:0097367
single-organism cellular process	P	1.11E-05	1.17	GO:0044763
cellular process	P	7.52E-05	1.10	GO:0009987
single-organism process	P	6.77E-04	1.12	GO:0044699
signaling	P	9.07E-04	1.30	GO:0023052
heterocyclic compound binding	F	1.11E-03	1.19	GO:1901363
organic cyclic compound binding	F	1.14E-03	1.18	GO:0097159
signal transduction	P	1.24E-03	1.32	GO:0007165
ion binding	F	1.24E-03	1.17	GO:0043167
cell communication	P	1.80E-03	1.29	GO:0007154
organelle organization	P	1.91E-03	1.36	GO:0006996
phosphate-containing compound metabolic process	P	1.91E-03	1.33	GO:0006796
protein kinase activity	F	2.65E-03	1.62	GO:0004672
single organism signaling	P	3.00E-03	1.29	GO:0044700
ATPase activity	F	3.48E-03	1.72	GO:0016887
cellular response to stimulus	P	3.78E-03	1.26	GO:0051716
phosphorus metabolic process	P	4.35E-03	1.31	GO:0006793
response to stimulus	P	4.96E-03	1.20	GO:0050896
protein serine/threonine kinase activity	F	5.13E-03	1.63	GO:0004674
multicellular organismal process	P	5.58E-03	1.22	GO:0032501

regulation of cellular process	P	5.58E-03	1.19	GO:0050794
cellular component organization	P	5.73E-03	1.25	GO:0016043
regulation of biological process	P	6.20E-03	1.17	GO:0050789
regulation of cell projection organization	P	6.21E-03	6.79	GO:0031344
Rho protein signal transduction	P	6.52E-03	2.29	GO:0007266
small GTPase binding	F	6.91E-03	19.02	GO:0031267
GTPase binding	F	7.38E-03	10.87	GO:0051020
actin filament-based process	P	8.11E-03	2.07	GO:0030029
calcium ion binding	F	8.36E-03	2.01	GO:0005509
single-multicellular organism process	P	8.45E-03	1.23	GO:0044707
protein phosphorylation	P	8.45E-03	1.50	GO:0006468
phosphotransferase activity, alcohol group as acceptor	F	8.49E-03	1.49	GO:0016773
nucleoside-triphosphatase activity	F	9.87E-03	1.39	GO:0017111
Ras protein signal transduction	P	1.01E-02	2.07	GO:0007265
regulation of Rho protein signal transduction	P	1.11E-02	2.26	GO:0035023
cell part	C	1.20E-02	1.08	GO:0044464
Ras GTPase binding	F	1.20E-02	17.66	GO:0017016
intracellular signal transduction	P	1.22E-02	1.42	GO:0035556
hydrolase activity, acting on acid anhydrides	F	1.27E-02	1.37	GO:0016817
actin cytoskeleton organization	P	1.30E-02	2.02	GO:0030036
phosphorylation	P	1.32E-02	1.45	GO:0016310
anatomical structure development	P	1.34E-02	1.23	GO:0048856
cellular component organization or biogenesis	P	1.50E-02	1.21	GO:0071840
hydrolase activity, acting on acid anhydrides, in phosphorus-containing anhydrides	F	1.71E-02	1.36	GO:0016818
pyrophosphatase activity	F	1.73E-02	1.36	GO:0016462
regulation of intracellular signal transduction	P	2.21E-02	1.76	GO:1902531
regulation of Ras protein signal transduction	P	2.21E-02	2.07	GO:0046578
single-organism organelle organization	P	2.26E-02	1.38	GO:1902589
regulation of response to stimulus	P	2.33E-02	1.50	GO:0048583
multicellular organismal development	P	2.51E-02	1.22	GO:0007275
regulation of cellular component	P	2.60E-02	1.71	GO:0051128

organization				
organophosphate catabolic process	P	3.05E-02	2.00	GO:0046434
single-organism developmental process	P	3.05E-02	1.20	GO:0044767
biological regulation	P	3.07E-02	1.14	GO:0065007
cytoskeleton organization	P	3.07E-02	1.49	GO:0007010
organelle part	C	3.08E-02	1.18	GO:0044422
motor activity	F	3.09E-02	2.30	GO:0003774
system development	P	3.47E-02	1.25	GO:0048731
intracellular organelle part	C	3.51E-02	1.19	GO:0044446
molecular function regulator	F	3.66E-02	1.52	GO:0098772
double-stranded RNA binding	F	3.98E-02	14.95	GO:0003725
chromosome	C	3.98E-02	1.56	GO:0005694
hydrolase activity, acting on acid anhydrides, catalyzing transmembrane movement of substances	F	3.99E-02	2.32	GO:0016820
transferase activity, transferring phosphorus-containing groups	F	3.99E-02	1.32	GO:0016772
plasma membrane	C	4.13E-02	1.69	GO:0005886
cellular protein modification process	P	4.26E-02	1.24	GO:0006464
protein modification process	P	4.26E-02	1.24	GO:0036211
regulation of signal transduction	P	4.41E-02	1.52	GO:0009966
nucleus	C	4.41E-02	1.22	GO:0005634
embryonic pattern specification	P	4.41E-02	2.50	GO:0009880
plasma membrane part	C	4.41E-02	1.75	GO:0044459
anatomical structure morphogenesis	P	4.66E-02	1.28	GO:0009653
regulation of small GTPase mediated signal transduction	P	4.77E-02	1.85	GO:0051056

[a] P, biological process; F, molecular function; C, cellular component

TABLE S12. Functional terms enriched among genes differentially methylated between castes or sexes relative to all DMGs.

Term	Category[a]	FDR	Fold enrichment	GO-ID
	Caste			
protein binding	F	3.60E-06	1.25	GO:0005515
purine ribonucleotide binding	F	2.21E-04	1.39	GO:0032555
ATP binding	F	3.16E-04	1.48	GO:0005524
protein complex	C	5.60E-04	1.40	GO:0043234
phosphate-containing compound metabolic process	P	1.33E-03	1.35	GO:0006796
intracellular membrane-bounded organelle	C	1.33E-03	1.18	GO:0043231
small molecule metabolic process	P	4.77E-03	1.35	GO:0044281
organonitrogen compound catabolic process	P	4.77E-03	1.80	GO:1901565
cell cycle phase	P	7.06E-03	2.41	GO:0022403
cellular amino acid metabolic process	P	9.05E-03	1.85	GO:0006520
single-organism catabolic process	P	1.09E-02	1.93	GO:0044712
cytoskeleton organization	P	1.68E-02	1.60	GO:0007010
cytoskeleton	C	2.22E-02	1.43	GO:0005856
nucleoplasm part	C	2.69E-02	1.94	GO:0044451
cytoplasmic part	C	3.24E-02	1.23	GO:0044444
aromatic compound catabolic process	P	4.07E-02	1.50	GO:0019439
organophosphate catabolic process	P	4.95E-02	1.57	GO:0046434

Term	Category[a]	FDR	Fold enichment	GO-ID
	Sex			
response to stimulus	P	5.25E-03	1.89	GO:0050896
nucleotide binding	F	1.51E-02	1.36	GO:0000166
regulation of biological process	P	2.41E-02	2.23	GO:0050789
cell communication	P	3.13E-02	1.33	GO:0007154
nucleoside-triphosphatase activity	F	4.88E-02	1.50	GO:0017111
anion binding	F	4.88E-02	1.33	GO:0043168
single organism signaling	P	4.88E-02	1.31	GO:0044700

[a] P, biological process; F, molecular function; C, cellular component

Table S13: Differentially methylated regions associated with transcription factor binding sites.

TFBS	Clover[a]		AME qvalue[b]		type call[c]	sex v caste[d]	Name	Znev gene ID[e]	log enrichment ratio[f]	
	cst	sex	cst	sex					caste	sex
p120	0	0	2.09E-06				Myb-interacting protein 120	Znev_00683	2.187	-3.188
Eip74EF	1	0	7.59E-06		caste	caste	Ecdysone-induced protein 74EF	Znev_00833	3.246	-0.403
fkh	1	0	1.00E-05		caste	caste	fork head	Znev_13477	3.053	NA
Ubx	1	0	3.93E-05		caste	caste	ultrabithorax	Znev_15380	0.783	NA
bab1	1	0	7.15E-06		caste	caste	bric a brac	Znev_03179	4.360	-0.796
br-Z2	1	1	4.47E-04	7.53E-03	both	caste	broad-Z2	Znev_09723	1.232	0.921
zen	1	0	2.51E-03		caste	caste	zerknullt	NA	1.468	-1.381
tll	1	0	3.24E-03		caste		tailless	Znev_12982	NA	-0.381
gt	1	0	3.44E-03		caste		giant	NA	3.526	0.619
en	1	0	5.28E-03		caste	caste	engrailed	Znev_15553	NA	NA
hkb	1	0	6.42E-03	1.72E-02	caste	caste	hucklebein	NA	7.430	6.490
exd	0	0	7.80E-03				extradenticle	Znev_12397	NA	NA
srp	1	0	2.94E-02		caste		serpent	Znev_02318	NA	NA
gsb	0	0	3.52E-02				gooseberry	NA	NA	2.204
hb	0	0	3.70E-02			caste	hunchback	Znev_01840	NA	NA
br-Z1	0	1	5.33E-04	5.33E-04	sex		broad-Z1	Znev_09723	NA	NA
SuH	1	1	7.34E-04	7.34E-04	sex		supressor of hairless	Znev_04163	-0.854	0.204
br-Z3	0	1	1.38E-03	1.38E-03	sex		broad-Z3	Znev_09723	NA	NA
Cf2-II	0	0	6.60E-03	6.60E-03	sex		Chorion factor 2	NA	NA	NA
nub	0	1	8.55E-03	8.55E-03	sex	sex	nubbin	Znev_14256	NA	2.204
vvl	1	1	1.15E-02	1.15E-02	sex		ventral veins lacking	Znev_11549	NA	NA
z	0	1	2.03E-02	2.03E-02	sex	sex	zeste	Znev_02821	0.070	4.374
brk	0	1	2.24E-02	2.24E-02	sex	sex	brinker	NA	NA	1.383
ems	1	0					empty spiracles	Znev_10939	0.002	1.028
ftz	1	0					fushi tarazu	Znev_18259	NA	NA
ap	1	0					apterous	Znev_18686	NA	NA
Dfd	1	0					Deformed	Znev_05733	NA	NA
cad	1	0					caudal	NA	NA	NA
bcd	1	1					bicoid	NA	NA	NA
grh	0	1					grainy head	Znev_13872	-2.232	-1.118
Med	1	0					Medea	Znev_02071	0.442	-0.052
tin	0	1				sex	tinman	NA	NA	0.089

ttk	0	1			sex	tram track	Znev_15196	-0.059	1.330

[a] binary values indicating whether caste (cst) or sex DMRs were significantly enriched for the given TFBS using the CLOVER program with two background control sets (see methods).

[b] qvalues from comparing caste (cst) and sex DMRs to control regions using the AME program

[c] value indicating if given TFBS is enriched among caste or sex DMRs – only TFBSs showing significant enrichment with *both* programs were considered enriched.

[d] value indicating if a given TFBS was enriched among either caste or sex DMRs when using the opposite as control sequences

[e] *Z. nevadensis* ortholog to the given *D. melanogaster* TF. NA indicates ortholog could not be found

[f] log2-transformed ratio of test:control hits for the given TFBS. NA indicates insufficient hits to produce ratio

Table S14: Top 10 significantly enriched motifs associated with differentially methylated regions of *Z. nevadensis* as determined by MEME *de novo* motif discovery.

Motif logo	width	e-value	Similarity hits[a]
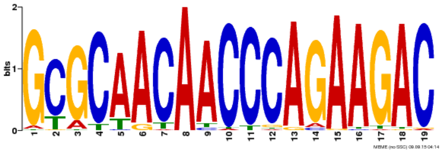	19	3.50E-260	Eip74EF
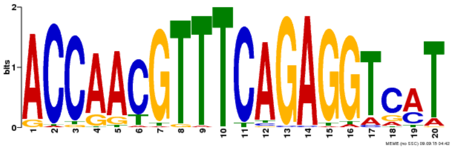	20	1.10E-227	Zne-mir-87-2-3p, Zne-mir-87-3-3p, Zne-mir-6012-5p, Zne-mir-282-3p, Zne-mir-9d-3p
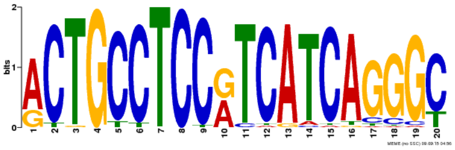	20	6.00E-200	dme-miR-313-5p, Zne-mir-34-3p
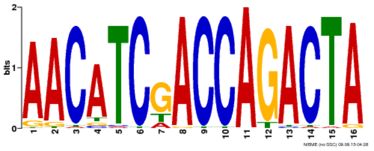	16	3.60E-141	NA
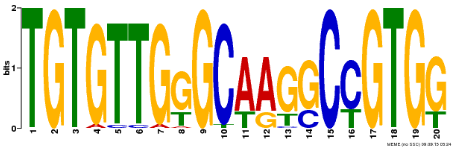	20	4.00E-103	NA
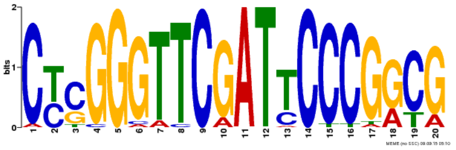	20	1.80E-98	dme-miR-4951-5p, dme-miR-4952-3p
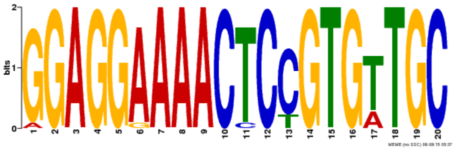	20	1.90E-79	dl
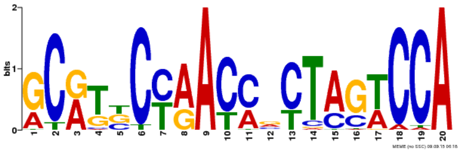	20	5.00E-61	bcd
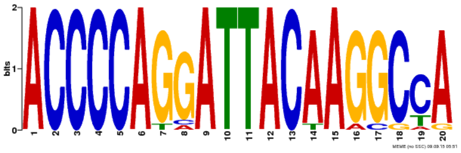	20	5.60E-59	Zne-mir-184-5p

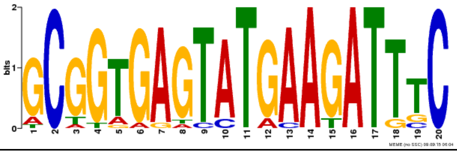	20	5.90E-48	dme-miR-1003-3p

[a]any significantly-similar (q-value < 0.25) miRNA or TFBS sequence motif.

Table S15: Differentially-methylated-region (DMR) miRNA homology tests for each *Z. nevadensis* miRNA showing homology to at least one DMR (89/190) as produced using the AME and FIMO tools from the MEME suite.

	CASTE				SEX			
miRNA	positive hits[a]	negative hits[a]	fold difference	AME FDR[b]	positive hits[a]	negative hits[a]	fold difference	AME FDR[b]
Zne-bantam-3p	0	3	0.470	ns	0	1	0.921	ns
Zne-bantam-5p	7	6	2.192	1.02E-07	14	4	6.448	0.03721
Zne-let-7-3p	0	0	NA	ns	0	0	NA	ns
Zne-let-7-5p	0	0	NA	ns	0	0	NA	ns
Zne-mir-1000-3p	2	3	1.409	ns	4	1	4.606	ns
Zne-mir-1000-5p	0	1	0.939	ns	0	0	NA	ns
Zne-mir-100-3p	0	0	NA	ns	0	0	NA	ns
Zne-mir-100-5p	0	0	NA	ns	0	0	NA	ns
Zne-mir-10-3p	0	2	0.626	ns	0	0	NA	ns
Zne-mir-10-5p	0	2	0.626	ns	0	0	NA	ns
Zne-mir-11-3p	4	0	9.394	ns	1	2	1.228	ns
Zne-mir-11-5p	0	0	NA	ns	1	1	1.842	ns
Zne-mir-1175-3p	3	2	2.505	ns	0	0	NA	ns
Zne-mir-1175-5p	0	1	0.939	ns	0	0	NA	ns
Zne-mir-12-3p	1	1	1.879	ns	0	3	0.461	ns
Zne-mir-124-3p	0	0	NA	ns	0	0	NA	ns
Zne-mir-124-5p	1	0	3.758	ns	6	2	4.299	ns
Zne-mir-125-3p	0	1	0.939	ns	0	0	NA	ns
Zne-mir-125-5p	10	0	18.788	0.0154	8	0	16.581	ns
Zne-mir-12-5p	0	0	NA	ns	0	4	0.368	ns
Zne-mir-133-3p	1	1	1.879	ns	0	1	0.921	ns
Zne-mir-133-5p	1	4	0.752	ns	3	0	7.370	ns
Zne-mir-137-3p	0	0	NA	ns	0	0	NA	ns
Zne-mir-137-5p	0	0	NA	ns	0	1	0.921	ns
Zne-mir-13a-1-3p	4	0	9.394	ns	2	0	5.527	ns
Zne-mir-13a-1-5p	0	1	0.939	ns	0	0	NA	ns
Zne-mir-13a-2-3p	4	0	9.394	ns	2	0	5.527	ns
Zne-mir-13a-2-5p	0	1	0.939	ns	0	0	NA	ns
Zne-mir-13b-3p	0	0	NA	ns	3	0	7.370	ns
Zne-mir-13b-5p	0	0	NA	ns	0	0	NA	ns
Zne-mir-1-3p	1	3	0.939	ns	0	3	0.461	ns
Zne-mir-14-3p	0	12	0.145	ns	22	0	42.375	ns
Zne-mir-14-5p	0	0	NA	ns	0	3	0.461	ns
Zne-mir-1-5p	4	0	9.394	ns	1	0	3.685	ns
Zne-mir-184-3p	0	0	NA	ns	0	0	NA	ns
Zne-mir-184-5p	1	0	1.879	7.18E-08	1	0	1.842	3.45E-05

Zne-mir-190-3p	0	0	NA	ns	0	2	0.614	ns
Zne-mir-190-5p	1	3	0.939	ns	0	0	NA	ns
Zne-mir-193-3p	1	0	3.758	ns	0	0	NA	ns
Zne-mir-193-5p	0	0	NA	ns	0	0	NA	ns
Zne-mir-210-3p	2	0	3.758	0.000942	4	0	9.212	ns
Zne-mir-210-5p	0	2	0.626	ns	1	0	3.685	ns
Zne-mir-219-3p	0	0	NA	ns	0	0	NA	ns
Zne-mir-219-5p	0	0	NA	ns	0	1	0.921	ns
Zne-mir-252-3p	0	0	NA	ns	0	1	0.921	ns
Zne-mir-252-5p	0	0	NA	ns	0	0	NA	ns
Zne-mir-263a-3p	24	0	45.092	1.11E-10	0	0	NA	ns
Zne-mir-263a-5p	2	0	5.637	ns	0	0	NA	ns
Zne-mir-263b-3p	0	1	0.939	ns	0	0	NA	ns
Zne-mir-263b-5p	0	0	NA	ns	2	1	2.764	ns
Zne-mir-275-3p	1	0	1.879	1.84E-11	9	0	16.581	0.000413
Zne-mir-275-5p	1	0	1.879	2.27E-05	0	0	NA	ns
Zne-mir-276-3p	0	0	NA	ns	0	0	NA	ns
Zne-mir-2765-3p	0	0	NA	ns	0	0	NA	ns
Zne-mir-2765-5p	0	0	NA	ns	1	0	3.685	ns
Zne-mir-276-5p	0	0	NA	ns	0	0	NA	ns
Zne-mir-277-3p	0	0	NA	ns	2	0	5.527	ns
Zne-mir-277-5p	3	0	5.637	6.23E-05	3	1	3.685	ns
Zne-mir-278-3p	0	0	NA	0.02103	1	0	3.685	ns
Zne-mir-278-5p	2	0	3.758	1.38E-09	3	0	5.527	0.01793
Zne-mir-2788-3p	0	2	0.626	ns	0	0	NA	ns
Zne-mir-2788-5p	0	0	NA	ns	0	2	0.614	ns
Zne-mir-2796-3p	5	0	9.394	0.04379	1	0	1.842	0.03191
Zne-mir-2796-5p	2	0	5.637	0.001209	0	0	NA	ns
Zne-mir-279a-3p	0	0	NA	ns	0	0	NA	ns
Zne-mir-279a-5p	1	1	1.879	1.76E-06	8	0	16.581	ns
Zne-mir-279c-3p	0	0	NA	ns	0	0	NA	ns
Zne-mir-279c-5p	4	0	7.515	5.97E-10	2	0	3.685	0.0167
Zne-mir-279d-3p	0	0	NA	ns	0	2	0.614	ns
Zne-mir-279d-5p	0	0	NA	ns	0	0	NA	ns
Zne-mir-281-3p	1	2	1.253	ns	2	1	2.764	ns
Zne-mir-281-5p	2	0	5.637	0.02979	0	1	0.921	ns
Zne-mir-282-3p	2	1	2.818	ns	0	0	NA	ns
Zne-mir-282-5p	3	2	2.818	7.37E-06	5	0	11.054	ns
Zne-mir-283-3p	0	0	NA	ns	0	0	NA	ns
Zne-mir-283-5p	0	1	0.939	ns	0	0	NA	ns
Zne-mir-29b-1-3p	0	0	NA	ns	0	0	NA	ns
Zne-mir-29b-1-5p	1	3	0.939	ns	0	2	0.614	ns
Zne-mir-2a-1-3p	0	1	0.939	ns	0	0	NA	ns

Zne-mir-2a-1-5p	2	0	5.637	ns	0	1	0.921	ns
Zne-mir-2a-2-3p	0	1	0.939	ns	0	0	NA	ns
Zne-mir-2a-2-5p	3	1	3.758	ns	1	0	3.685	ns
Zne-mir-2a-3-3p	0	1	0.939	ns	0	0	NA	ns
Zne-mir-2a-3-5p	10	0	18.788	1.39E-11	1	0	3.685	ns
Zne-mir-2a-4-3p	0	1	0.939	ns	0	0	NA	ns
Zne-mir-2a-4-5p	0	0	NA	ns	8	1	8.291	ns
Zne-mir-2b-3p	0	0	NA	ns	0	0	NA	ns
Zne-mir-2b-5p	4	0	9.394	ns	0	0	NA	ns
Zne-mir-3049-3p	2	0	5.637	0.004251	1	0	1.842	0.04929
Zne-mir-3049-5p	4	0	7.515	4.41E-06	0	0	NA	ns
Zne-mir-305-3p	0	0	NA	ns	0	0	NA	ns
Zne-mir-305-5p	0	0	NA	ns	0	0	NA	ns
Zne-mir-306-3p	0	1	0.939	ns	6	0	12.897	ns
Zne-mir-306-5p	0	0	NA	ns	0	0	NA	ns
Zne-mir-307-3p	1	0	3.758	ns	0	0	NA	ns
Zne-mir-307-5p	0	1	0.939	ns	0	2	0.614	ns
Zne-mir-31-3p	1	0	3.758	ns	2	0	5.527	ns
Zne-mir-315-3p	1	1	1.879	ns	0	0	NA	ns
Zne-mir-315-5p	0	0	NA	ns	0	0	NA	ns
Zne-mir-31-5p	1	0	3.758	ns	0	0	NA	ns
Zne-mir-316-3p	11	3	5.637	ns	0	2	0.614	ns
Zne-mir-316-5p	2	0	3.758	3.33E-05	0	0	NA	ns
Zne-mir-317-3p	2	0	3.758	ns	0	3	0.461	ns
Zne-mir-317-5p	1	0	1.879	0.01643	0	0	NA	ns
Zne-mir-33-3p	3	4	1.503	ns	5	0	11.054	ns
Zne-mir-33-5p	0	1	0.939	ns	0	0	NA	ns
Zne-mir-34-3p	55	0	103.337	8.97E-13	19	0	35.005	0.000468
Zne-mir-34-5p	0	0	NA	ns	0	0	NA	ns
Zne-mir-3477-3p	0	0	NA	ns	0	0	NA	ns
Zne-mir-3477-5p	13	14	1.754	ns	7	7	1.842	ns
Zne-mir-375-3p	0	1	0.939	ns	0	0	NA	ns
Zne-mir-375-5p	0	0	NA	ns	2	0	5.527	ns
Zne-mir-3770-3p	2	6	0.805	ns	0	0	NA	ns
Zne-mir-3770-5p	0	0	NA	ns	0	1	0.921	ns
Zne-mir-6012-3p	0	0	NA	ns	3	0	5.527	0.01583
Zne-mir-6012-5p	267	16	31.353	3.03E-15	5	9	1.105	ns
Zne-mir-71-1-3p	1	0	3.758	ns	8	0	16.581	ns
Zne-mir-71-1-5p	0	0	NA	ns	0	0	NA	ns
Zne-mir-71-2-3p	1	0	3.758	ns	8	0	16.581	ns
Zne-mir-71-2-5p	0	0	NA	ns	0	0	NA	ns
Zne-mir-7-3p	1	0	3.758	ns	0	5	0.307	ns
Zne-mir-750-3p	2	0	5.637	ns	0	0	NA	ns

Zne-mir-750-5p	1	0	3.758	ns	6	0	11.054	0.03797
Zne-mir-7-5p	0	2	0.626	ns	0	2	0.614	ns
Zne-mir-79-3p	0	0	NA	ns	2	0	5.527	ns
Zne-mir-79-5p	0	0	NA	ns	0	0	NA	ns
Zne-mir-8-3p	1	0	3.758	ns	0	0	NA	ns
Zne-mir-8-5p	1	1	1.879	ns	0	0	NA	ns
Zne-mir-87-1-3p	14	4	6.576	1.43E-05	1	2	1.228	ns
Zne-mir-87-1-5p	1	0	3.758	ns	0	0	NA	ns
Zne-mir-87-2-3p	13	3	6.576	1.43E-05	1	2	1.228	ns
Zne-mir-87-2-5p	0	0	NA	1.85E-05	0	3	0.461	ns
Zne-mir-927a-3p	0	1	0.939	0.004438	0	0	NA	ns
Zne-mir-927a-5p	0	0	NA	ns	0	1	0.921	ns
Zne-mir-927b-3p	0	0	NA	ns	0	0	NA	ns
Zne-mir-927b-5p	0	0	NA	ns	1	6	0.526	ns
Zne-mir-929-3p	0	0	NA	ns	1	0	3.685	ns
Zne-mir-929-5p	0	0	NA	ns	0	0	NA	ns
Zne-mir-92a-3p	1	0	1.879	0.01317	0	0	NA	ns
Zne-mir-92a-5p	0	0	NA	ns	0	0	NA	ns
Zne-mir-92b-3p	2	0	3.758	0.002017	0	0	NA	ns
Zne-mir-92b-5p	0	0	NA	ns	0	2	0.614	ns
Zne-mir-92c-3p	0	0	NA	ns	2	0	5.527	ns
Zne-mir-92c-5p	0	0	NA	ns	0	0	NA	ns
Zne-mir-932-1-3p	0	1	0.939	ns	0	5	0.307	ns
Zne-mir-932-1-5p	0	0	NA	ns	0	0	NA	ns
Zne-mir-932-2-3p	0	1	0.939	ns	0	5	0.307	ns
Zne-mir-932-2-5p	0	0	NA	ns	0	0	NA	ns
Zne-mir-965-3p	1	0	3.758	ns	0	0	NA	ns
Zne-mir-965-5p	0	1	0.939	ns	0	1	0.921	ns
Zne-mir-971-3p	0	0	NA	ns	0	1	0.921	ns
Zne-mir-971-5p	0	0	NA	ns	0	0	NA	ns
Zne-mir-980-1-3p	0	0	NA	ns	0	0	NA	ns
Zne-mir-980-1-5p	4	0	7.515	3.24E-05	2	0	5.527	ns
Zne-mir-980-2-3p	0	0	NA	ns	0	0	NA	ns
Zne-mir-980-2-5p	1	0	3.758	3.24E-05	2	0	5.527	ns
Zne-mir-981-3p	0	0	NA	0.0077	0	0	NA	ns
Zne-mir-981-5p	3	0	5.637	1.57E-12	1	0	3.685	1.09E-05
Zne-mir-989-1-3p	0	1	0.939	ns	0	0	NA	ns
Zne-mir-989-1-5p	0	0	NA	ns	0	0	NA	ns
Zne-mir-989-2-3p	0	1	0.939	ns	1	0	3.685	ns
Zne-mir-989-2-5p	0	0	NA	ns	0	0	NA	ns
Zne-mir-993-3p	1	0	3.758	ns	0	2	0.614	ns
Zne-mir-993-5p	2	0	5.637	ns	0	0	NA	ns
Zne-mir-995-3p	0	0	NA	ns	0	0	NA	ns

Zne-mir-995-5p	1	0	1.879	0.02062	2	0	5.527	ns
Zne-mir-998-3p	2	0	3.758	ns	0	0	NA	ns
Zne-mir-998-5p	1	0	1.879	3.56E-05	7	0	12.897	0.006444
Zne-mir-9a-3p	0	0	NA	ns	0	0	NA	ns
Zne-mir-9a-5p	0	0	NA	ns	0	0	NA	ns
Zne-mir-9c-3p	0	1	0.939	ns	0	3	0.461	ns
Zne-mir-9c-5p	0	0	NA	ns	0	0	NA	ns
Zne-mir-9d-3p	0	0	NA	ns	0	0	NA	ns
Zne-mir-9d-5p	0	1	0.939	ns	0	0	NA	ns
Zne-mir-iab-4-3p	0	0	NA	ns	1	0	3.685	ns
Zne-mir-iab-4-5p	0	0	NA	ns	2	2	1.842	ns
Zne-mir-iab-8-3p	0	0	NA	ns	1	0	3.685	ns
Zne-mir-iab-8-5p	0	0	NA	ns	1	0	3.685	ns
**Totals**	**556**	**136**	**7.681**		**222**	**110**	**3.718**	

[a]number of DMRs showing significant (FDR < 0.1) homology to the given miRNA sequence for Caste and Sex differing DMRs.

[b]results of testing whether DMR sequences show higher representation of the given mature miRNA than surrounding control methylated regions.

Table S16: miRNA-hit-containing DMRs are generally not 3'-associated. For all DMRs that contain a significant miRNA profile hit the number falling within each genic feature is given, along with the proportion of all such DMRs this feature contains.

`Feature[a]	count	proportion
upstream	34	0.052
exon	158	0.244
intron	401	0.619
downstream	55	0.085
**Total**	648	

[a]Upstream: -1.5kb – ATG; downstream STOP - +1.5kb.

Table S17. Gene ontology enrichment associated with genes containing miRNA-hitting DMRs relative to all DMR-containing genes.

GO Term	Category[a]	FDR	fold enrichment	GO ID
regulation of small GTPase mediated signal transduction	P	3.70E-05	5.36	GO:0051056
regulation of intracellular signal transduction	P	7.70E-04	3.85	GO:1902531
response to stimulus	P	1.50E-03	1.59	GO:0050896
regulation of cell communication	P	5.10E-03	2.72	GO:0010646
regulation of signal transduction	P	5.10E-03	2.84	GO:0009966
regulation of response to stimulus	P	6.50E-03	2.55	GO:0048583
single organism signaling	P	6.70E-03	1.69	GO:0044700
GTPase regulator activity	F	6.70E-03	5.44	GO:0030695
regulation of Rho protein signal transduction	P	6.70E-03	4.53	GO:0035023
signaling	P	6.70E-03	1.67	GO:0023052
nucleoside-triphosphatase regulator activity	F	7.80E-03	4.77	GO:0060589
molecular function regulator	F	7.80E-03	2.70	GO:0098772
cell communication	P	8.10E-03	1.66	GO:0007154
signal transduction	P	9.40E-03	1.70	GO:0007165
Rho protein signal transduction	P	9.40E-03	4.21	GO:0007266
regulation of Ras protein signal transduction	P	1.20E-02	4.03	GO:0046578
cellular response to stimulus	P	1.20E-02	1.60	GO:0051716
anion binding	F	1.40E-02	1.68	GO:0043168
synapse organization	P	1.40E-02	6.64	GO:0050808
intracellular signal transduction	P	1.90E-02	2.08	GO:0035556
enzyme activator activity	F	2.00E-02	4.31	GO:0008047
anatomical structure development	P	2.00E-02	1.57	GO:0048856
guanyl-nucleotide exchange factor activity	F	2.60E-02	5.08	GO:0005085
nucleoside phosphate binding	F	3.10E-02	1.59	GO:1901265
system development	P	3.30E-02	1.63	GO:0048731
imaginal disc pattern formation	P	3.80E-02	5.37	GO:0007447
SWI/SNF superfamily-type complex	C	4.90E-02	16.11	GO:0070603
skeletal muscle fiber development	P	1.20E-01	10.25	GO:0048741
nerve development	P	1.50E-01	16.92	GO:0021675

[a]P, biological process; F, molecular function; C, cellular component
